# The superior performance of silica gel supported nano zero-valent iron for simultaneous removal of Cr (VI)

**DOI:** 10.1038/s41598-022-26612-1

**Published:** 2022-12-23

**Authors:** Eslam Salama, Mahmoud Samy, Hassan Shokry, Gehan El-Subruiti, Asmaa El-Sharkawy, Hesham Hamad, Marwa Elkady

**Affiliations:** 1 Environment and Natural Materials Research Institute (ENMRI), City of Scientific Research and Technological Applications (SRTA-City), New Borg El-Arab City, Alexandria, 21934 Egypt; 2 Department of Public Works Engineering, Faculty of Engineering, Mansoura University, Mansoura, 35516 Egypt; 3 Environmental Engineering Department, Egypt-Japan University of Science and Technology (E-JUST), New Borg El-Arab City, Alexandria, Egypt; 4 Electronic Materials Research Department, Advanced Technology and New Materials Research Institute, City of Scientific Research and Technological Applications (SRTA-City), New Borg El-Arab City, Alexandria, 21934 Egypt; 5 Chemistry Department, Faculty of Science, Alexandria University, Ibrahimia, Alexandria, Egypt; 6 Fabrication Technology Research Department, Advanced Technology and New Materials Research Institute, City of Scientific Research and Technological Applications (SRTA-City), New Borg El-Arab City, Alexandria, 21934 Egypt; 7 Chemical and Petrochemical Engineering Department, Egypt-Japan University of Science and Technology (E-JUST), New Borg El-Arab City, Alexandria, 21934 Egypt

**Keywords:** Environmental sciences, Chemistry, Materials science, Nanoscience and technology, Nanoscale materials

## Abstract

Pure nano zero-valent iron (NZVI) was fabricated under optimum conditions based on material production yield and its efficiency toward acid blue dye-25 decolorization. The optimum prepared bare NZVI was immobilized with two different supports of silica and starch to fabricate their composites nanomaterials. The three different prepared zero-valent iron-based nanomaterials were evaluated for removal of hexavalent chromium (Cr(VI)). The silica-modified NZVI recorded the most outstanding removal efficiency for Cr(VI) compared to pristine NZVI and starch-modified NZVI. The removal efficiency of Cr(VI) was improved under acidic conditions and decreased with raising the initial concentration of Cr(VI). The co-existence of cations, anions, and humic acid reduced Cr(VI) removal efficiency. The removal efficiency was ameliorated from 96.8% to 100% after adding 0.75 mM of H_2_O_2_. The reusability of silica-modified NZVI for six cycles of Cr(VI) removal was investigated and the removal mechanism was suggested as the physicochemical process. Based on Langmuir isotherm, the maximal Cr(VI) removal capacity attained 149.25 mg/g. Kinetic and equilibrium data were efficiently fitted using the pseudo-second-order and Langmuir models, respectively confirming the proposed mechanism. Diffusion models affirmed that the adsorption rate was governed by intraparticle diffusion. Adsorption thermodynamic study suggested the spontaneity and exothermic nature of the adsorption process. This study sheds light on the technology that has potential for magnetic separation and long-term use for effective removal of emerging water pollutants.

## Introduction

Chromium ions and dyes can be released with the industrial effluents of various industries such as steel, electroplating, textile, tannery, leather, paper, food and cosmetics^1,2^. The presence of chromium ions and dyes in surface and groundwater has negative impacts on living organisms due to their high toxicity, carcinogenicity, and biorecalcitrance^3,4^. Chromium is a heavy metal that frequently exists in two forms (trivalent [Cr(III)] and hexavalent [Cr(VI)])^5^. Cr(VI) is featured by its higher toxicity, solubility, and mobility compared to Cr(III)^6^. The existence of Cr(VI) ions in the human body can result in severe risks such as skin disease, diabetes and respiratory problems^7^. Moreover, it can destroy RNA and DNA^8^. The permissible concentration of Cr(VI) in the industrial effluents before the discharge to surface water is less than 0.05 mg L^-1^ according to international standards^9^. Moreover, dyes are characterized by their high color strength affecting negatively the photosynthesis process^10^. Therefore, the industrial effluents containing Cr(VI) or/and dyes have to receive an effective treatment before their release into aquatic systems.

Various techniques such as photocatalysis, chemical precipitation, membrane filtration, coagulation and biological processes have been employed for the removal of Cr(VI) and dyes^11–16^. However, the aforementioned techniques have some defects such as the low removal performance, high cost and generation of secondary contaminants^17–19^. The adsorption process combined with chemical reduction is a promising technique due to its simplicity, low-cost and high removal performance towards various pollutants^20^. Moreover, the secondary pollutants generated via this process are limited^21^. Recently, nano zero-valent iron (NZVI) has been evaluated as an adsorbent owing to its inexpensiveness, high removal capacity, availability, facile preparation and high surface area^22,23^. Furthermore, NZVI can reduce dyes to less toxic compounds and Cr(VI) to Cr(III)^24^. The preparation of NZVI using different reducing agents and iron precursors under different reaction times can greatly affect the yield and removal performance of NZVI. Therefore, the optimization of the preparation of NZVI under the aforementioned factors was conducted based on the yield and the removal performance of acid blue-25 dye. Despite the aforementioned excellent characteristics of NZVI, it can easily aggregate because of its magnetic properties and van der Waals forces which adversely affect the reactivity and reducibility performance of NZVI^25^. Additionally, oxidation of NZVI can frequently take place leading to the formation of a corrosion layer on its surface obstructing the extended use of NZVI^26^.

The aforementioned issues can be solved by supporting the NZVI surface on different organic or inorganic supports such as starch, chitosan, dextran, silica, biochar, bentonite and kaolinite^27–31^. The introduction of these supports onto the NZVI surface can ameliorate the dispersibility, de-agglomeration, stability, reactivity, and reducibility and avert the oxidation of NZVI surface^32^. On the other hands, several studies for overcoming the drawbacks of Fe^0^ have investigated by various methods like doping the surface by nobel metals (e.g.; Cu, Ni, Au and Ag) and coating with Mg(OH)_2_ overcoming the poor mobility and low suspension stability of bare-Fe^0^ ^33,34^.

Therefore, NZVI was either supported on inorganic silica gel (Si) or organic starch (St) supports to fabricate zero-valent based composite nanomaterial. These composites were evaluated for the adsorption and reduction of Cr(VI) due to the low-cost, high stability and green nature of these stabilizers. Kumari et al. (2020) synthesized NZVI modified with starch for the removal of chromium^31^. The results showed that the removal efficiency of chromium was lower than 50% in the case of pure NZVI compared to nearly 65% using NZVI supported on starch. Furthermore, SEM images confirmed the reduction of nanoparticles aggregation after the modification by starch. Wang et al. (2021) synthesized zero-valent iron coated with silica for the decolorization of acid red 73 dye^35^. Acid red 73 with an initial concentration of 100 mg L^-1^ was completely removed after 120 min using 2 g/L of the silica modified NZVI. Additionally, the performance of NZVI can be improved by adding an oxidizing agent (e.g., H_2_O_2_) due to its ability to enhance the corrosion of NZVI^36^. Moreover, reactive radicals can be produced via the activation of H_2_O_2_ by the active sites on the NZVI surface and the reactive species can contribute to the reduction of Cr(VI)^37^.

In this study, the optimization of the preparation process of NZVI was conducted. Furthermore, the optimum prepared NZVI based on the production yield and it is affecting on dye decolorization was modified with silica gel or starch and the synthesized nanomaterials were characterized. The performance of the synthesized nanomaterials for the adsorption and chemical reduction of Cr(VI) was studied. Furthermore, the adsorption integrated with the reduction of Cr(VI) by the synthesized materials under different operating parameters such as pH and initial pollutant concentration was investigated. The effects of the co-existence of anions, cations, and natural organic matter on the removal efficiency of Cr(VI) were explored. Additionally, isotherms, thermodynamics, and kinetics of the adsorption process were studied. The reusability performance of the synthesized materials was evaluated, and the removal mechanism was explained. This is the first study to gain better insights into the role of silica supported Fe^0^ on the boosting removal of Cr (VI) ions after optimizing the synthetic procedures of Fe^0^.

## Results and discussion

### Optimization of the preparation process of NZVI

**Figure 1:**
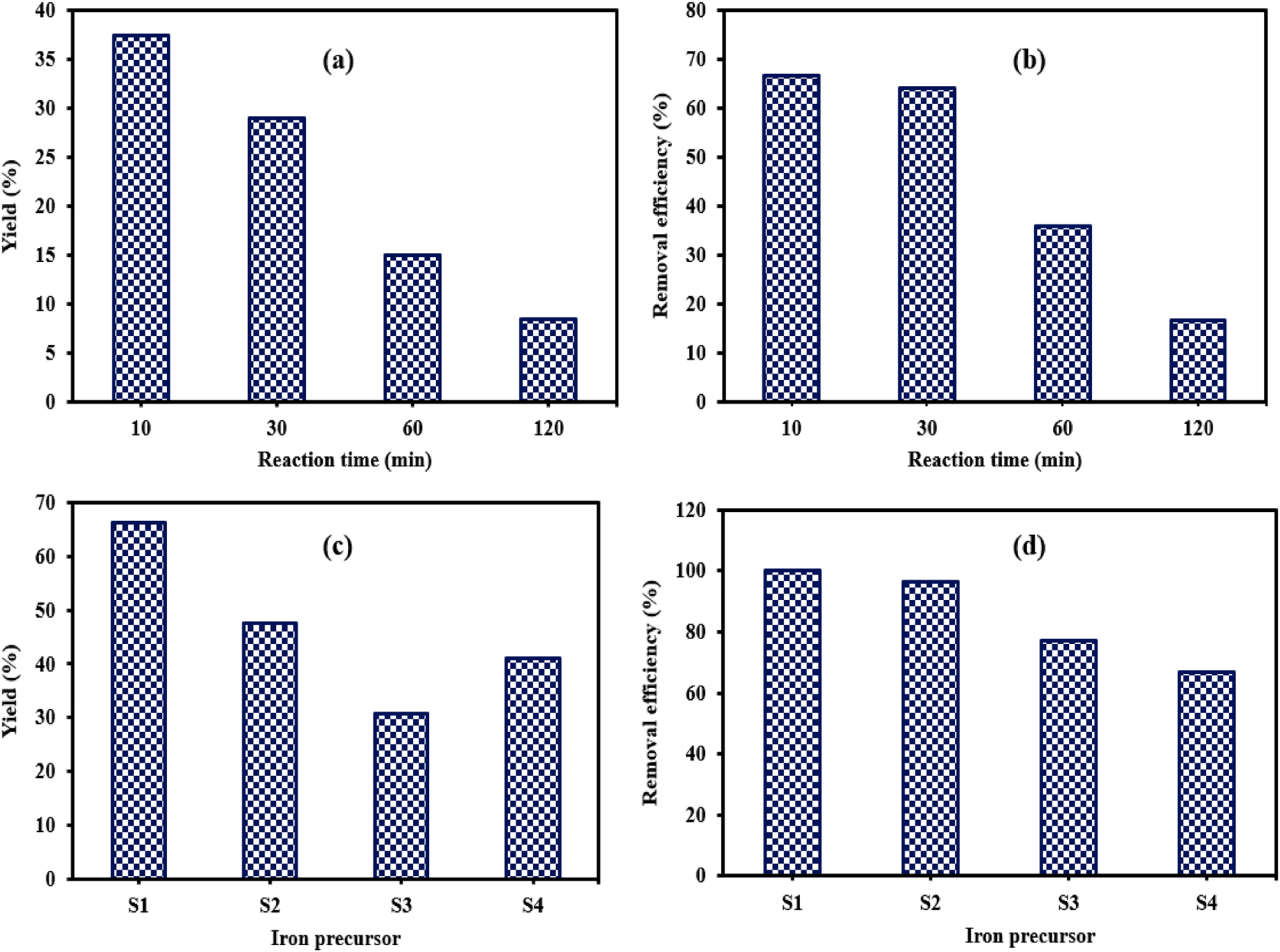
Effect of reaction time on (**a**) NZVI yield (%) and (**b**) removal efficiency of acid blue-25 (%) and Effect of iron salt on (**c**) NZVI yield (%) and (**d**) removal efficiency of acid blue-25 (%). (S1: FeCl_2_.4H_2_O, S2: FeCl_2_, S3: FeCl_3_.6 H_2_O, S4: FeSO_4_.7H_2_O, dye concentration = 50 mg L^-1^, NZVI dose = 0.1 g, solution volume = 100 mL, agitating time = 60 min, agitation rate = 200 rpm).

The effects of reaction time, reducing agents, and iron precursors on the preparation process of NZVI were optimized based on NZVI yield and the performance of the prepared NZVI towards the decolorization of acid blue-25. The preparation process of NZVI requires a suitable reaction time to obtain metallic iron nanoparticles, so the preparation of NZVI was conducted at reaction times of 10, 30, 60 and 120 min using NaBH_4_ and FeCl_2_.4H_2_O as a reducing agent and iron precursor, respectively. The yield of NZVI and removal efficiency of dye decreased with raising the reaction time as shown in Fig. 1a,b, respectively. The NZVI yield decreased from 37.38% to 8.4% by extending the time from 10 to 120 min and the removal efficiency of the dye decreased from 66.7 to 16.5%. The extension of reaction time may increase the tendency of NZVI surface oxidation and the formation of iron oxides. It was noticed that the increase in time resulted in the raising of rusting. So, the optimum reaction time for NZVI production was considered as 10 min.

The reducing agent has a significant role in the preparation process of NZVI. To specify the most proper reducing agent for NZVI synthesis, NaBH_4_, N_2_H_4_, NaOH and NH_4_OH with 4 M were used as reducing agents following the previously mentioned synthesis procedures of NZVI.

In the case of using NaBH_4_, a black magnetic precipitate was produced. However, NaOH and N_2_H_4_ produced a brown precipitate showing lower magnetic properties. In the case of NH_4_OH, a non-magnetic green color precipitate was formed. The results showed that N_2_H_4_, NaOH and NH_4_OH could not effectively reduce the iron salt to its zero state. Therefore, NaBH_4_ was the most suitable reducing agent for the preparation of NZVI.

Four iron salts were used (FeCl_2_.4H_2_O, FeCl_2_, FeSO_4_.7H_2_O and FeCl_3_.6H_2_O) to specify the best iron salt for the preparation of NZVI based on the yield (%) and dye removal efficiency (%) as demonstrated in Fig. 1c,d. The preparation process was conducted using the optimum reaction time and reducing agent. The results indicated that FeCl_2_.4H_2_O attained the highest yield (66.35%) and removal efficiency of dye (99.9%) compared to other salts. Moreover, the results confirmed that the preparation of NZVI by Fe(II) was better than that produced using Fe(III). Therefore, the optimum iron salt for NZVI production was FeCl_2_.4H_2_O.

In the synthesis of NZVI, the economic cost into consideration for the need of the clean water, especially in developing countries. Hence, in the calculations for removal efficiency, it is important to calculate the cost-effectiveness index of the nZVI-synthesis parameters, especially with the optimization of different reducing agents due to the cost of reducing agent is so high. In addition, the relevant cost and energy consumption should be taken into consideration for a practical optimization^38^.

### Physico-chemical characteristics of the synthesized nanomaterials

**Figure 2:**
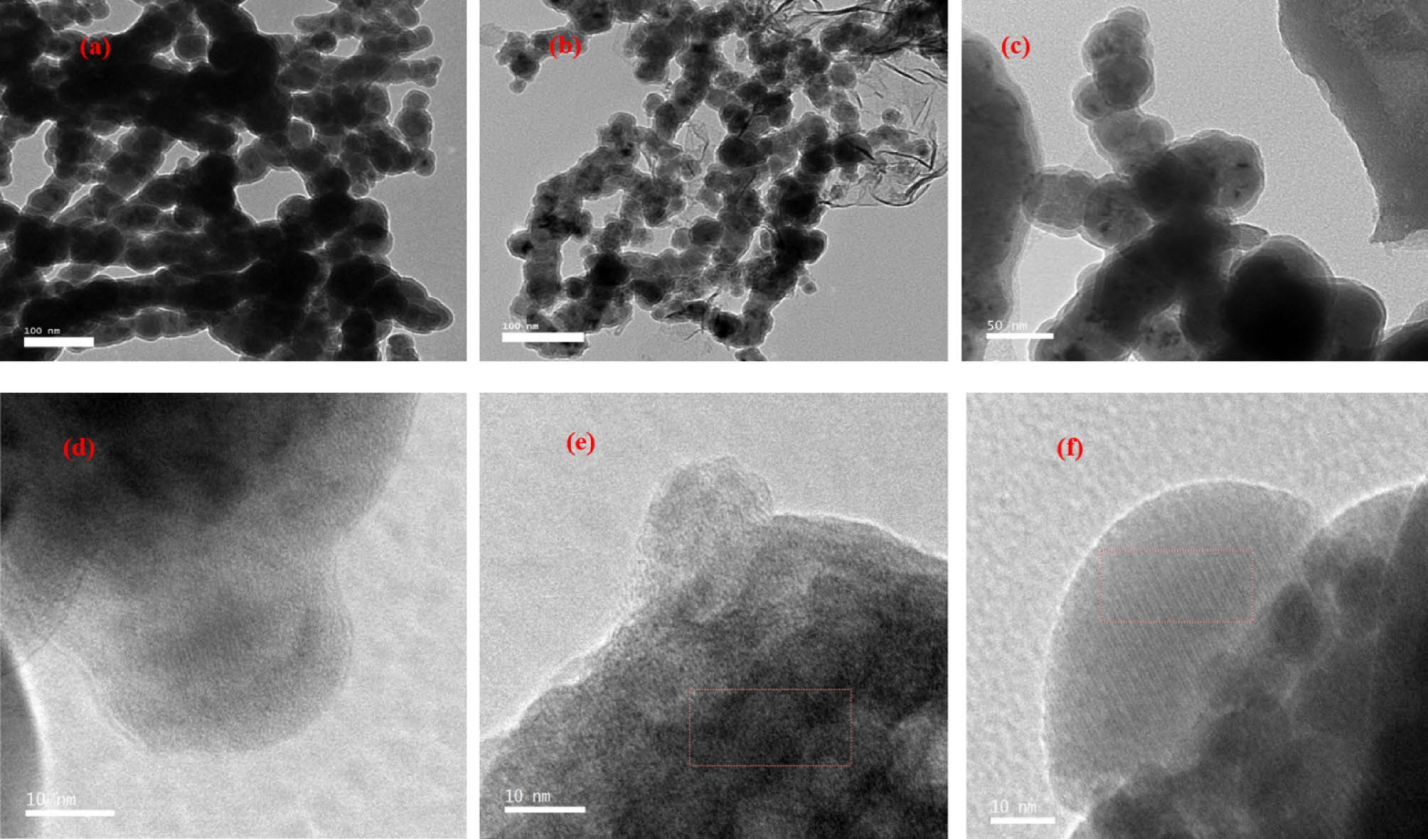
TEM images of (**a**) NZVI, (**b**) NZVI-St and (**c**) NZVI-Si; and high-resolution TEM images (**d**) NZVI, (**e**) NZVI-St and (**f**) NZVI-Si.

TEM image of bare NZVI (Fig. 2a) showed a chain-like agglomeration of nanoparticles, whereas the aggregation of nanoparticles in the case of NZVI-St and NZVI-Si as shown in (Fig. 2b,c) was lower confirming the role of different supports as stabilizers in providing the required repulsive forces between nanoparticles. The nanoparticles had a spherical shape with low tendency of aggregation for the composite materials with a particle size ranged from 20 to 50 nm in the case of pure NZVI and modified NZVI. The grey area in TEM images of NZVI-Si and NZVI-St is attributed to silica gel and starch supporting materials confirming the excellent support of NZVI on silica gel and starch. Moreover, high-resolution TEM images in Fig. 2d–f insured the excellent support of NZVI on silica gel or starch.

**Figure 3:**
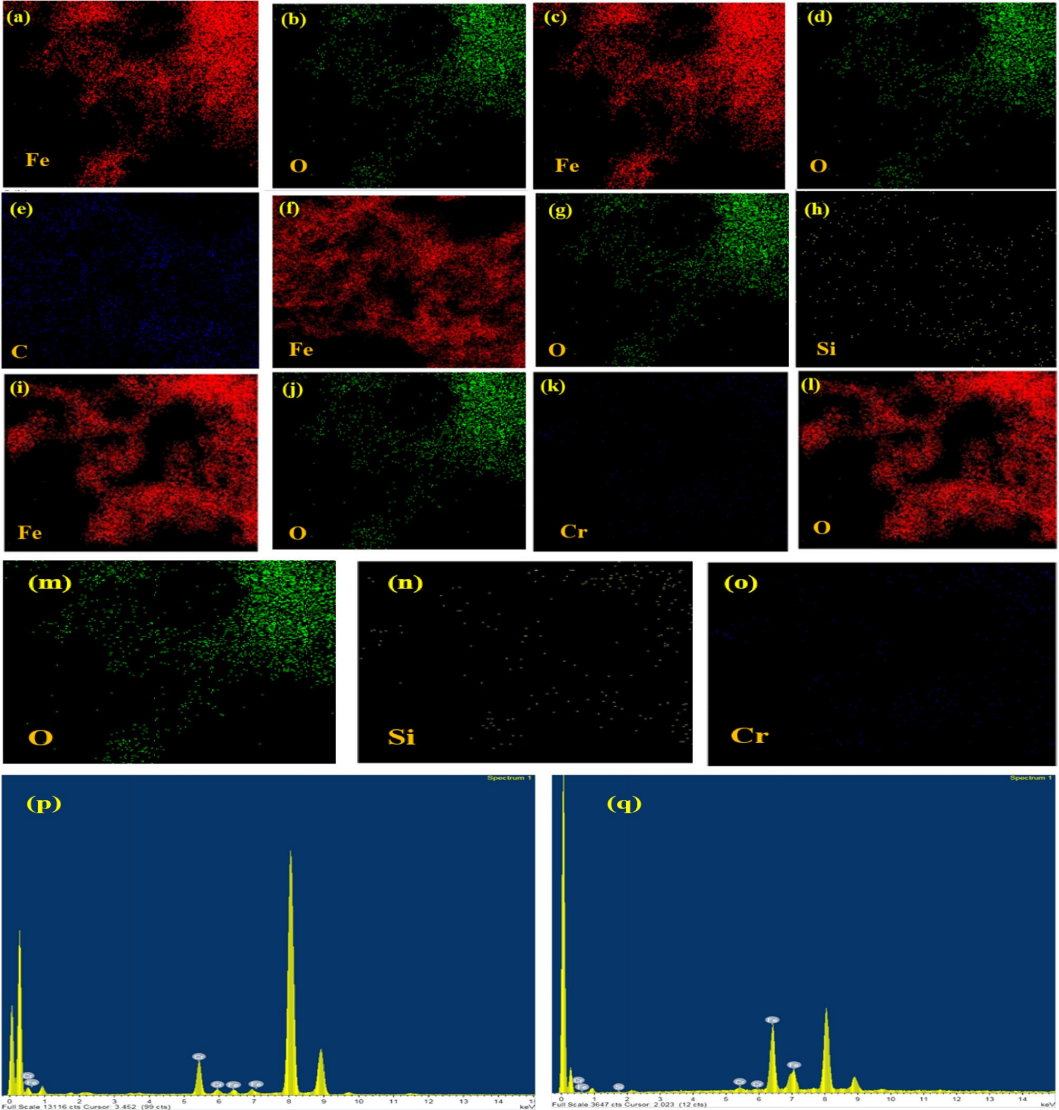
EDX elemental mapping of NZVI (**a**) Fe and (**b**) O; NZVI-St (**c**) Fe, (**d**) O and (**e**) C; NZVI-Si (**f**) Fe, (**g**) O and (**h**) Si; NZVI after Cr(VI) removal (**i**) Fe, (**j**) O and (**k**) Cr; NZVI-Si after Cr(VI) removal (**l**) Fe, (**m**) O, (**n**) Si and (**o**) Cr and EDS pattern after Cr(VI) removal of (**p**) NZVI and (**q**) NZVI-Si.

To investigate the chemical composition of the synthesized nanomaterials, the EDS pattern in Fig. S1a–c showed the growth of Fe and O in the case of NZVI confirming the partial oxidation of NZVI, whereas the presence of Fe, O and Si in the case of NZVI-Si and Fe, O and C for NZVI-St was affirmed. Moreover, EDS elemental mapping in Fig. 3a–h reconfirmed the chemical composition of the synthesized nanomaterials. Additionally, the EDS and elemental mapping analyses were performed after the removal of Cr(VI) and the results demonstrated the introduction of Cr to the chemical composition in the case of NZVI and NZVI-Si as shown in Fig. 3i–q which insures the adsorption of Cr(VI) on the surface of the adsorbent. SAED patterns in Fig. S2 showed the crystallinity of the synthesized materials.

**Figure 4:**
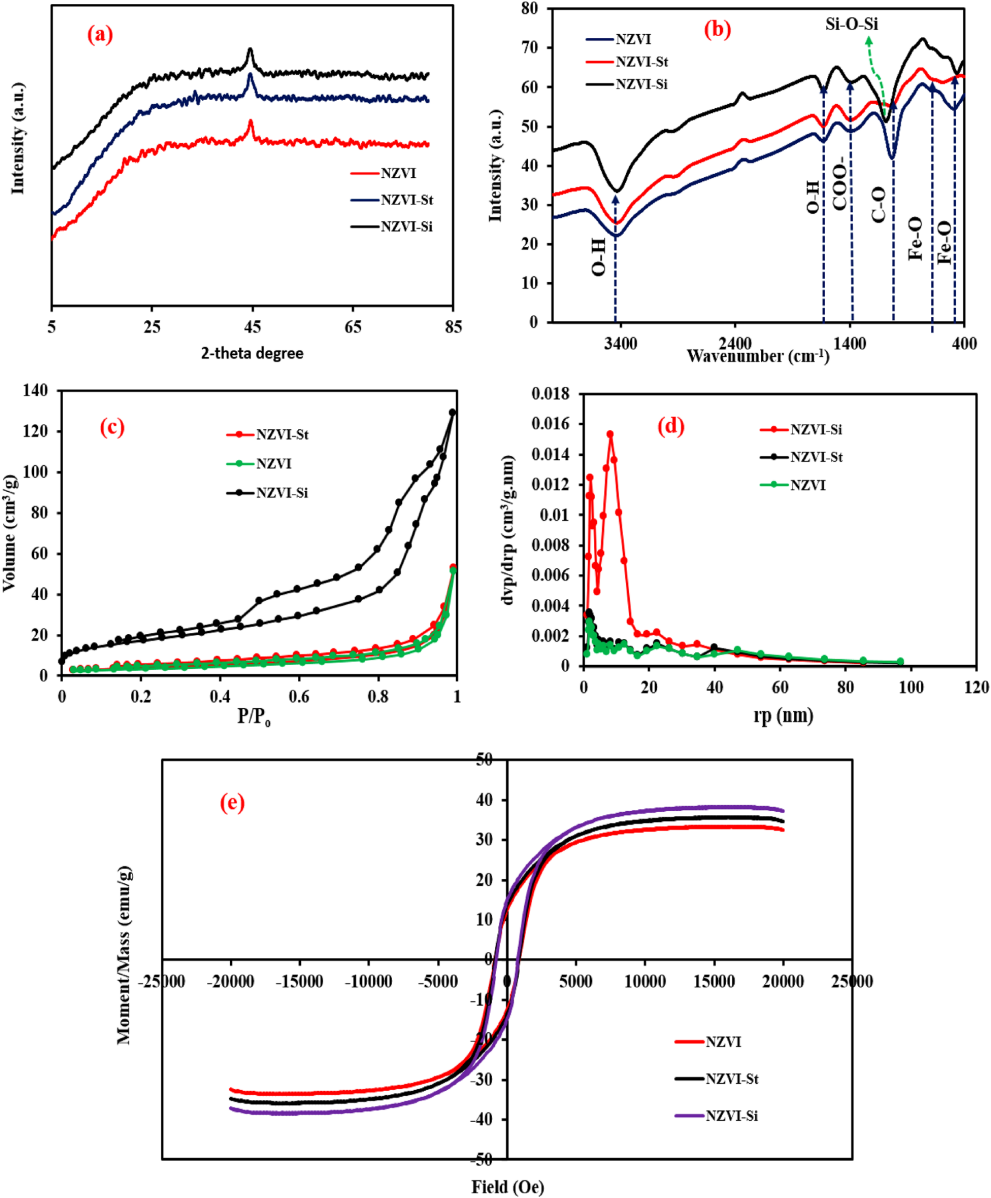
(**a**) XRD patterns; (**b**) FTIR spectra; (**c**) adsorption–desorption isotherms; (**d**) pore size distribution and (**e**) VSM of NZVI, NZVI-St and NZVI-Si.

Figure 4a show the XRD patterns of NZVI, NZVI-St and NZVI-Si. The significant peak at around 45^o^ is ascribed to the NZVI diffraction plane (110) (JCPDS, file No. 87–0722), whereas there is no peak nearly 35° is imputed to the (311) diffraction plane of Fe_3_O_4_ , confirming there is no or rare formation of the iron oxide layer on the NZVI surface during the preparation process^39,40^. Chemical structures of the three zero-valent based nanomaterials were confirmed using FTIR analysis. The bands at around 1618 and 3423 cm^−1^ are ascribed to bending and vibration of the O–H bond owing to the adsorbed water molecules on the adsorbent’s surface in the case of NZVI, whereas the bands at 3417 and 1635 cm^−1^ in the case of NZVI-Si are ascribed to the presence of O–H bond besides the existence of stretching vibrations of Si–O–Si group at 1095 cm^−1^ as depicted in Fig. 4b ^41,42^. Regarding NZVI-St, the band at 2912 cm^−1^ is allocated to the CH_2_OH group in the starch which confirms the excellent support of NZVI on starch and the bands at 1624 and 3424 cm^−1^ are imputed to the adsorbed water molecules on the surface^41^. The bands around 1030 cm^−1^ and 1380 cm^−1^ are ascribed to the C-O and COO^-^ bonds respectively which are presence at NZVI, NZVI-Si and NZVI-St^43^. The bands at nearly 484, 476 and 565 cm^−1^ are allocated to the stretching vibrations of Fe–O in the case of NZVI and its composites NZVI-Si and NZVI-St, respectively^44^. As shown in Fig. 4c, both NZVI and NZVI-St showed type-II adsorption isotherm, while NZVI-Si showed type-IV isotherm. The porous texture of NZVI-Si is different from NZVI and NZV-St, confirming the characteristic effect of the mesoporous structure of silica in NZVI-Si. In addition, NZVI-Si presents a developed few micropore structures that strongly favors high surface area values compared to those for NZVI and NZVI-St. The calculated surface areas of the three prepared materials NZVI, NZVI-St and NZVI-Si were 12, 14, and 60 m^2^/g, respectively, confirming the significant enhancement of surface area after the support on silica that resulted to the excellent adsorption performance of NZVI-Si. In Fig. 4d, NZVI-Si has a higher mesopore volume than NZVI-St, resulting in the higher removal of pollutants via pore. The magnetic saturation values (Fig. 4e) were 38.312, 33.44, and 35.756 emu/g, in the case of NZVI, NZVI-St, and NZVI-Si, respectively, which elucidate the ferromagnetic characteristics of the synthesized nanomaterials. However, the reduction of magnetic saturation values in the case of NZVI-St and NZVI-Si was due to the non-magnetic properties of the supporting materials either starch or silica. Although their magnetic characteristics are weaker than those of NZVI, it is stated that samples have outstanding magnetic properties to facilitate separation.

**Figure 5:**
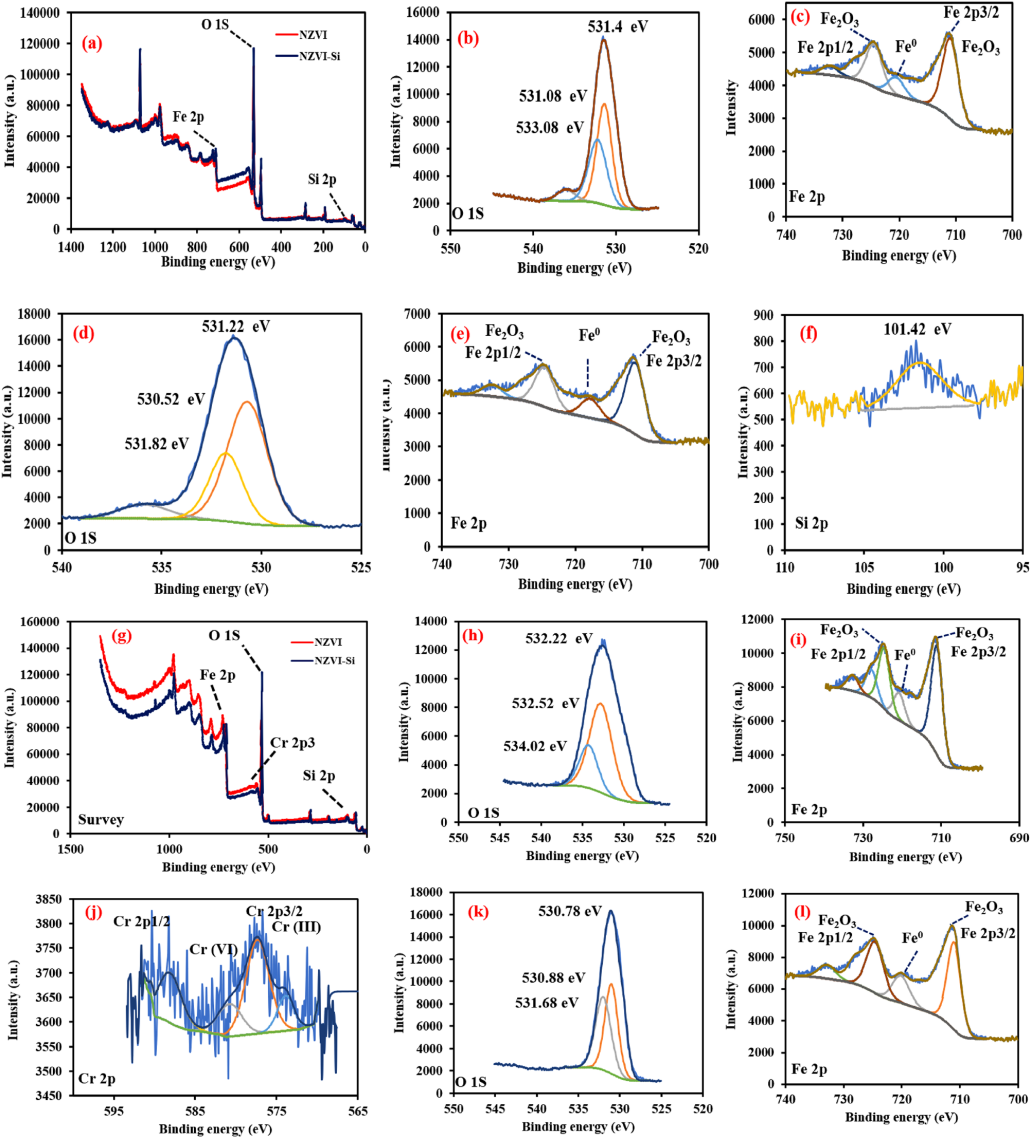

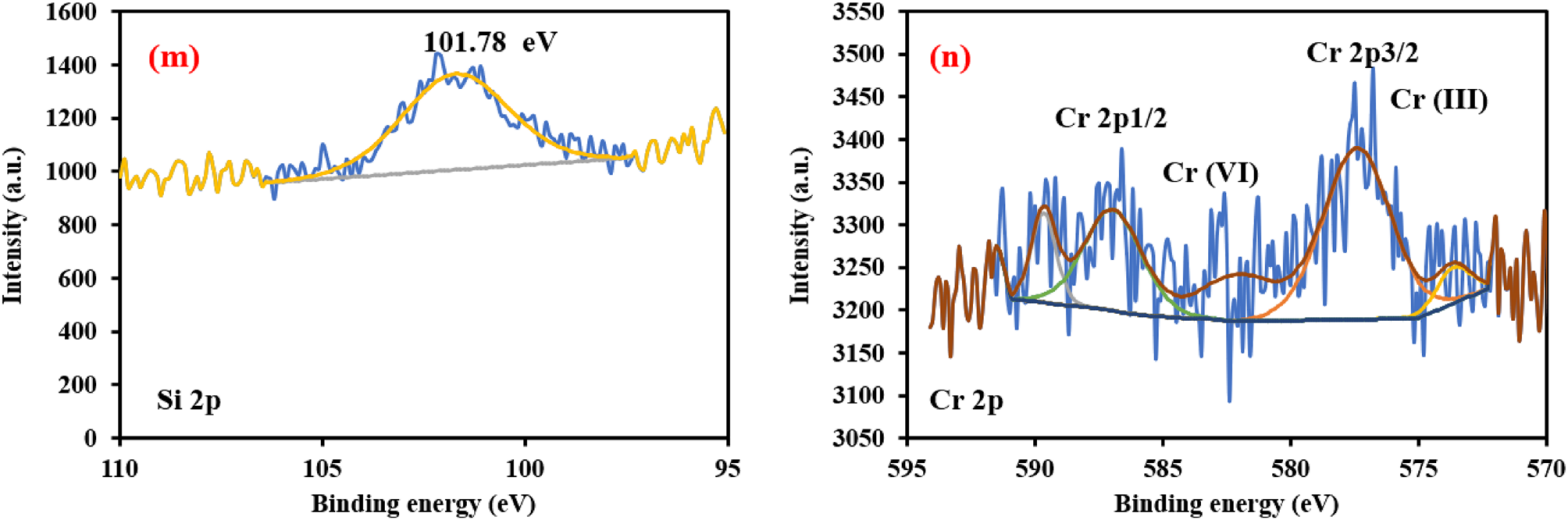
(**a**) XPS survey of NZVI and NZVI-Si, (**b**) O _1 s_ spectra and (**c**) Fe _2p_ spectra of NZVI, (**d**) O _1 s_ spectra, (**e**) Fe _2p_ spectra and (**f**) Si _2p_ spectra of NZVI-Si, (**g**) XPS survey of NZVI and NZVI-Si after Cr(VI) removal, (**h**) O _1 s_ spectra, (**i**) Fe _2p_ spectra and (**j**) Cr _2p_ spectra of NZVI, (**k**) O _1 s_ spectra, (**l**) Fe _2p_ spectra, (**m**) Si _2p_ and (**n**) Cr _2p_ of NZVI-Si.

XPS spectra affirmed the presence of Fe and O and Fe, O and Si in the case of NZVI and NZVI-Si, respectively as shown in Fig. 5a. The peaks at nearly 531.40, 531.08 and 533.08 eV are ascribed to O_1s_ in the case of pure NZVI, whereas the peaks at around 531.22, 530.52 and 531.82 eV are attributed to O _1S_ in the case of NZVI-Si^45^ as depicted in Fig. 5b,d. The peaks at about 723.68, 710.66 eV and 719.76 eV in Fig. 5c,e are imputed to Fe 2p_1/2_, Fe 2p_3/2_ and Fe^0^, respectively which reconfirms the formation of the iron oxide layer in the case of NZVI and NZVI-Si^46^. The peak at nearly 101.56 eV is ascribed to the Si 2p which affirms the excellent support of NZVI on Si (Fig. 5f)^26^.

The existence of the Cr 2p peak revealed that the Cr(VI) was successfully trapped on the NZVI-Si (Fig. 5g). The high resolution XPS spectra were recorded after the Cr(VI) removal process and insignificant shifts in the binding energies were observed due to the complexation between Cr(VI) and the adsorbents’ surface as shown in Fig. 5g–m. The peak at 581.9 eV is ascribed to Cr(VI) which affirms the adsorption of Cr(VI) on the surface (Fig. 5n). Additionally, the peaks at 577.68 and 588.23 eV are attributed to Cr 2p_3/2_ and Cr 2p_1/2_ of Cr(III), respectively in the case of NZVI and confirming the reduction of Cr(VI) to Cr(III) after adsorption on the materials surfaces^40^. The existence of Fe^0^ peak after Cr(VI) removal suggests the high reusability capability of both NZVI-Si and NZVI-St.

### Effect of pH and initial Cr(VI) concentration on the removal performance

**Figure 6:**
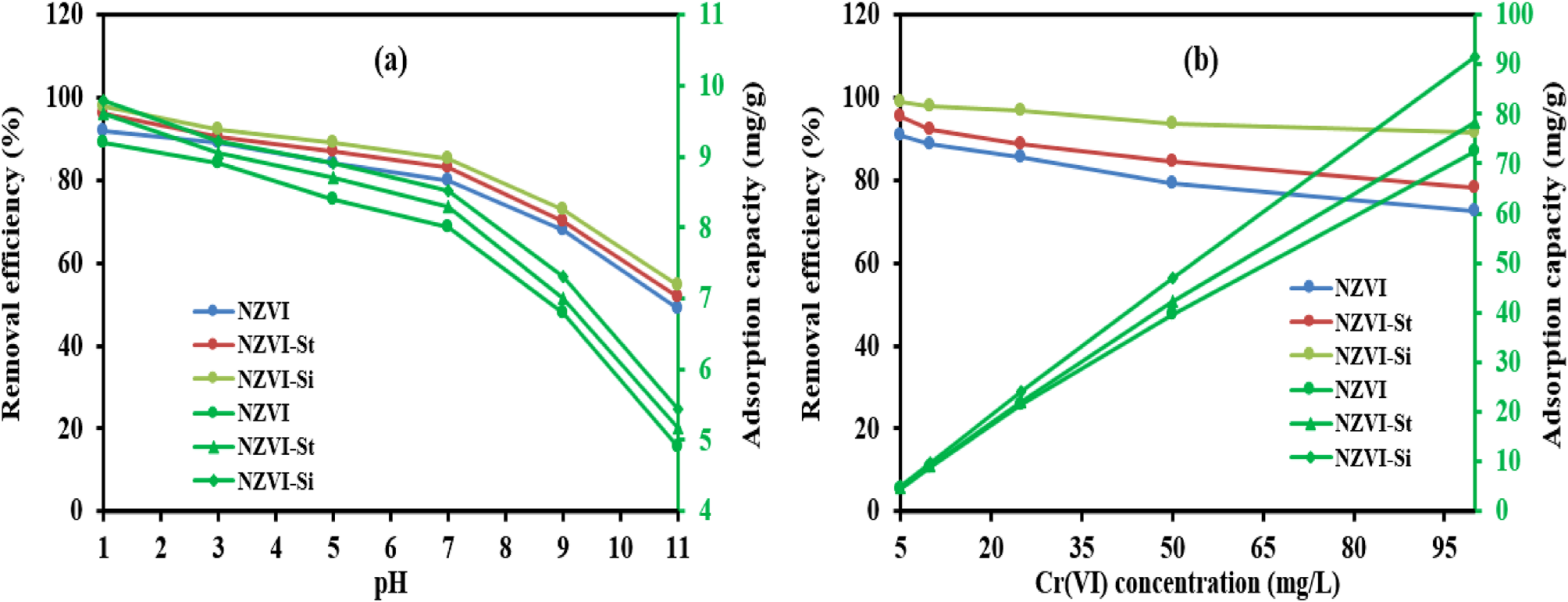
(**a**) Effect of pH and (**b**) Effect of initial Cr(VI) on the removal efficiency of Cr(VI) and adsorption capacity of synthesized materials.

Figure 6a portrays the effect of pH on the removal efficiency of Cr(VI) and the adsorption capacity of the synthesized nanomaterials at an adsorbent dose of 0.1 g/100 mL, initial Cr(VI) concentration of 10 mg L^-1^, contact time of 120 min and temperature of 20 °C. The removal efficiencies decreased from 91.9 to 49%, 96 to 51.8% and 98 to 54.5% by raising the pH from 1 to 11 using NZVI, NZVI-St and NZVI-Si, respectively. Kumari et al. (2020) reported the improvement of Cr(VI) removal at low pH value using starch modified NZVI^33^. At low pH values (1-3), NZVI and modified NZVI showed high removal performance and adsorption capacity. The synthesized nanomaterials can be easily corroded at low pH values which contributes to the dissolution of Fe^0^ and the production of hydrogen and Fe^2+^ (secondary reductants) as shown in Eqs. (1,2)^4,47^. Moreover, low pH values can participate in the dissolution of the passivation layer formed on the NZVI surface which provides fresh active sites^48^. The point of zero charge (PZC) of NZVI, NZVI-Si and NZVI-St were estimated to be 7.2, 8.1 and 7.8, respectively. At a pH value lower than the PZC, the adsorbents’ surface charge becomes positive. Therefore, the removal efficiency was enhanced due to the attractive forces between positively charged NZVI, NZVI-Si and NZVI-St and Cr(VI) anions, because Cr(VI) exists in the form of HCrO_4_^-^ and CrO_4_^2-^ over a wide pH range^47^.

The increase in pH decreased the removal efficiency of Cr(VI) and the adsorption capacity of NZVI, NZVI-St and NZVI-Si because of the repulsive forces between NZVI, NZVI-Si and NZVI-St and Cr(VI) anions^4^. Furthermore, iron hydroxides can be easily formed on the adsorbents’ surface in alkaline conditions, and they can block the active sites which decreases the reactivity of NZVI, NZVI-Si and NZVI-St^48^. Moreover, the iron oxides on the adsorbents’ surface can de-accelerate the electron transfer from the adsorbent to sorbent^49^. The removal efficiency was enhanced in alkaline conditions using modified NZVI, as silanol groups in the support can be separated and provide protons that maintain the pH and prevents the passivation^4^.

Figure 6b shows the effect of initial Cr(VI) concentration on the adsorption capacity of NZVI, NZVI-Si and NZVI-St and the removal efficiency of Cr(VI). NZVI-Si and NZVI-St showed higher removal performance compared to pristine NZVI at different initial Cr(VI) concentrations due to the reduction of aggregation of nanoparticles after the support on silica and starch as explained in the characterizations section. Moreover, the introduction of these supports can reduce the oxidation of the adsorbents’ surface and inhibit the formation of passivation layer which facilitates the electron transfer between NZVI and Cr(VI). Also, the amelioration of the removal performance of Cr(VI) in the case of modified NZVI was due to the increase in the surface area.

The increase of adsorption capacity from 4.95 to 91.5 mg g^−1^ by raising the initial concentration of Cr(VI) from 5 to 100 mg L^−1^ at pH 1, contact time of 120 min, the temperature of 20 °C and NZVI-Si dose of 0.1 g/100 mL was owing to the ameliorated driving forces between Cr(VI) and reactive sites. On the other hand, the removal efficiency of Cr(VI) went down from 90.8 to 72.4%, 95.6 to 78.3% and 99 to 91.5%, respectively with the increase of initial Cr(VI) concentration from 5 to 100 mg L^−1^ using NZVI, NZVI-St and NZVI-Si, respectively. In the case of low concentrations of Cr(VI), the active sites on the NZVI, NZVI-Si and NZVI-St are adequate for the reduction and adsorption of Cr(VI). However, in the case of high concentrations of Cr(VI), the reactive sites are not enough to adsorb the high number of Cr(VI) ions which inhibits the appropriate contact between the adsorbents and Cr(VI) ions^50^. Moreover, the formation of passivation layer on the adsorbents’ surface can be accelerated in the case of high Cr(VI) concentration which reduces the reactivity of the synthesized adsorbents with Cr(VI) and prevents the electron transfer from NZVI surface to Cr(VI)^51,52^. Zhou et al. (2022) reported the same trend during the removal of Cr(VI) by a modified NZVI^53^. Due to the superiority of NZVI-Si for Cr(VI) adsorption is over NZVI-St and NZVI, NZVI-Si. It was selected to investigate the remaining parameters affected on Cr(VI) removal as well as the experimental data of Cr(VI) removal using NZVI-Si were fitted using adsorption kinetic, isotherm and thermodynamic models.

1 $$ {\text{2HCrO}}_{{4}}^{ - } + {\text{ 3Fe}}^{0} + {\text{ 14 H}}^{ + } \to {\text{2 Cr}}\left( {{\text{III}}} \right) \, + {\text{ 3 Fe}}\left( {{\text{II}}} \right) \, + {\text{ 8 H}}_{{2}} {\text{O}} $$

2 $$ {\text{HCrO}}_{{4}}^{ - } + {\text{ 3 Fe}}\left( {{\text{II}}} \right) \, + {\text{ 7 H}}^{ + } \to {\text{Cr}}\left( {{\text{III}}} \right) \, + {\text{ 3 Fe}}\left( {{\text{III}}} \right) \, + {\text{ 4 H}}_{{2}} {\text{O}} $$

### Effect of co-existing cations, anions and humic acid on the removal efficiency

**Figure 7:**
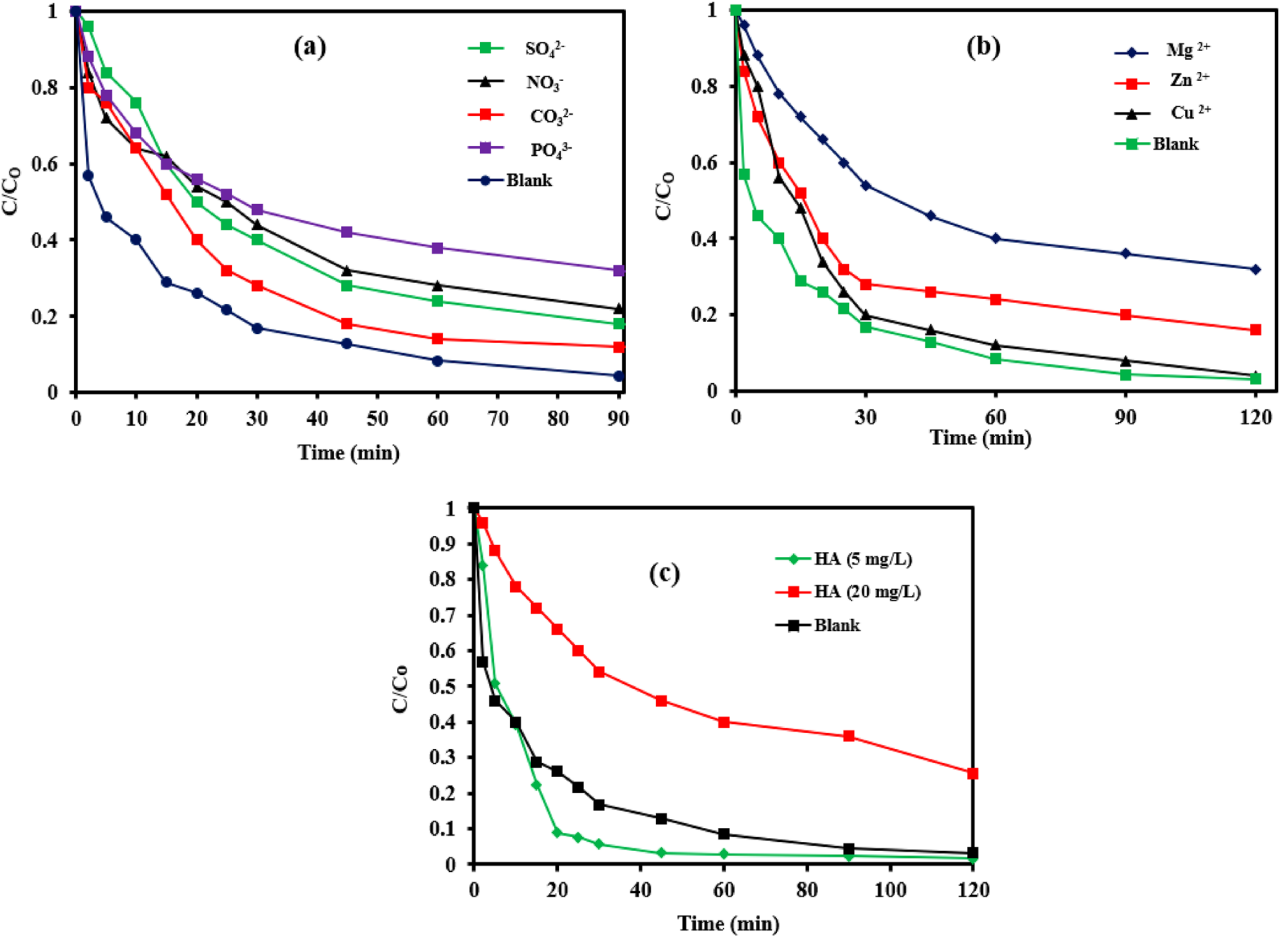
(**a**) Effect of co-existing (**a**) anions, (**b**) cations and (**c**) humic acid on the removal efficiency of Cr(VI) by NZVI-Si.

The influences of the presence of anions, cations and natural organic matter such as humic acid (HA) on the Cr(VI) removal efficiency onto NZVI-Si were explored to simulate the real application, as real wastewater contains a mixtures of species such as inorganic ions and natural organic matter. Fig. 7a shows the effect of the presence of anions (e.g., NO_3_^-^, CO_3_^2-^, SO_4_^2-^, PO_4_^3-^) with a concentration of 40 mg L^−1^ on the removal efficiency of Cr(VI). The removal efficiencies of Cr(VI) were 83.6%, 79.6%, 90.2% and 75.6% after adding SO_4_^2-^, NO_3_^-^, CO_3_^2-^ and PO_4_^3-^, respectively compared to 96.8% in the case of no anions at NZVI-Si dose of 0.1 g/100 mL, initial Cr(VI) concentration of 25 mg L^−1^, contact time of 120 min and temperature of 20 °C. In spite of the improvement of the ionic strength after adding the aforementioned anions, they can compete with Cr(VI) and occupy the binding sites which de-accelerate the removal of Cr(VI)^53^. Furthermore, anions such as CO_3_^2-^ and PO_4_^3-^ can form inner-sphere complexes with iron (oxy) hydroxides and the formed complexes can block the active sites reducing the removal efficiency^54^.

The investigation of the influence of the existence of cations such as Mg^2+^, Zn^2+^ and Cu^2+^on the removal efficiency of Cr(VI) was carried out as shown in Fig. 7b at NZVI-Si dose of 0.1 g/100 mL, initial Cr(VI) concentration of 25 mg L^−1^, contact time of 120 min, cations concentrations of 40 mg L^−1^ and temperature of 20 °C. The removal efficacy of Cr(VI) was 96.8% in the case of no-cations, whereas the addition of Mg^2+^ and Zn^2+^ decreased the removal efficiencies to 68% and 84%, respectively. Mg^2+^ and Zn^2+^ ions can occupy the reactive sites instead of Cr(VI) which decreases the removal efficiency of Cr(VI). On the other hand, the removal efficiency of Cr(VI) was 96% after adding Cu^2+^. The electron transfer and corrosion of Fe^0^ can be improved owing to the formed bimetallic surface after adding Cu^2+^. The enhancement of electron transfer and corrosion after the addition of Cu^2+^ can overweigh the negative effect of the occupation of binding sites by Cu^2+^ ions leading to high removal efficiency approximately the same as the blank sample. Chen et al. (2016) stated the improvement of the removal of hexachlorobenzene after Cu^2+^ addition using NZVI composited with activated carbon^54^.

The removal efficiency of Cr(VI) was enhanced to 98.4% in the presence of 5 mg L^−1^ of HA compared to 96.8% in the absence of HA as shown in Fig. 7c at NZVI-Si dose of 0.1 g/100 mL, initial Cr(VI) concentration of 25 mg L^−1^, contact time of 120 min, and temperature of 20 °C. The presence of humic acid can propel the removal of Cr(VI) due to its ability to act as an electron shuttle enhancing the electron transfer between NZVI and Cr(VI)^51^. Moreover, the formation of NOM agglomeration on the NZVI-Si surface can ameliorate the adsorption of Cr(VI) on the NZVI-Si surface. Lv et al. (2013) reported an increase in the Cr(VI) removal efficiency after adding 5 mg L^−1^ of HA^55^. The raising of HA concentration to 20 mg L^−1^ decreased the removal efficiency of Cr(VI) to 74.4%. At high concentrations of humic acid, it can compete with Cr(VI) for the adsorption on the NZVI-Si surface. Moreover, HA can react with (Fe^2+^/Fe^3+^) decreasing the number of binding sites. Beside complexes may be formed between humic acid and iron ions which inhibit the formation of corrosion products involved in the removal of Cr(VI)^36^.

### Effect of the addition of H_2_O_2_ on the removal of Cr(VI) by NZVI-Si

**Figure 8:**
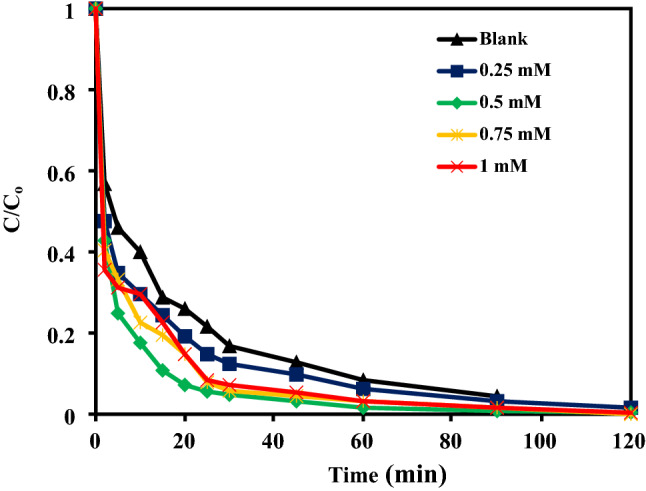
(**a**) Effect of the addition of different concentrations of H_2_O_2_ on the removal efficiency of Cr(VI) at NZVI-Si dose of 0.1 g/100 mL, initial Cr(VI) concentration of 25 mg/L, contact time of 120 min, and temperature of 20 °C.

The addition of 0.25, 0.5 and 0.75 mM of H_2_O_2_ to the adsorption system ramped the removal efficiency to 98.4, 99.6% and 100%, respectively, whereas it was 96.8% without adding H_2_O_2_ as shown in Fig. 8. The generated radicals after the activation of H_2_O_2_ by NZVI-Si can produce reducing conditions that can contribute to the reduction of Cr(VI) to Cr(III). Moreover, the introduction of H_2_O_2_ can speed up the corrosion of NZVI-Si^36^. The increase of H_2_O_2_ concentration can support the generation of more radicals, accelerate the NZVI-Si corrosion, and the reduction of Cr(VI)^36^. However, the increase of H_2_O_2_ to 1 mM decreased the removal efficiency due to its scavenging impact towards generated radicals at elevated concentrations^37^. Moreover, the reduced Cr(III) can be reconverted to Cr(VI) at high H_2_O_2_ concentrations^36^.

### Adsorption kinetics

**Figure 9:**
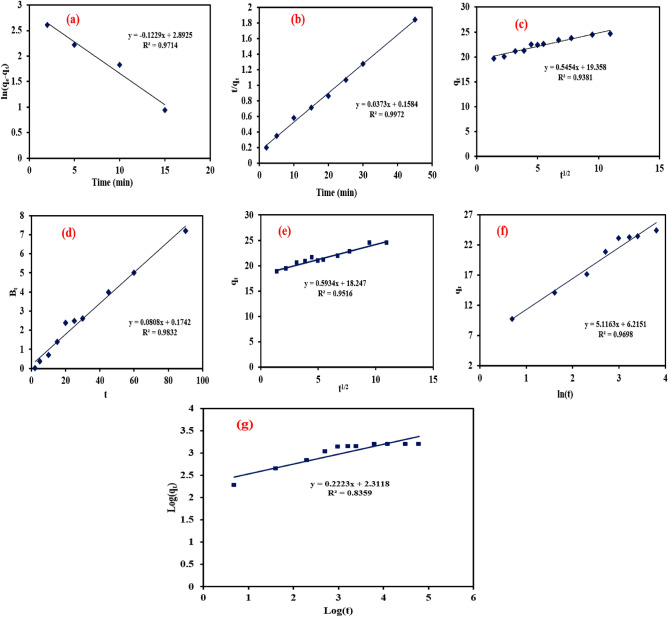
Adsorption kinetics of Cr(VI) (**a**) first-order model, (**b**) second-order model, (**c**) intraparticle diffusion, (**d**) Boyd model, (**e**) parabolic diffusion model, (**f**) Elovich model and (**g**) power function model.

The pseudo-first-order rate constant and coefficient of determination (R^2^) were estimated via the linear plot of ln (q_e_-q_t_) versus time (t) as shown in Fig. 9a. The R^2^ was high (0.97); however, there was a significant difference between experimental q_e_ (23.39 mg g^−1^) and obtained q_e_ (18.04 mg g^−1^) from the pseudo-first-order model. Therefore, the pseudo-first-order model was not efficient to describe the adsorption of Cr(VI) on the NZVI-Si surface. Regarding the pseudo-second-order model, the model constants were estimated from the slope and the intercept of the linear relation between t/q_t_ and t as depicted in Fig. 9b. The significance of the pseudo-second-order model was affirmed by the high R^2^ (0.9972) and the slight difference between experimental q_e_ (23.39 mg g^−1^) and calculated q_e_ (26.8 mg g^−1^) from the model. The intraparticle diffusion model constants and R^2^ were estimated by the linear plot of q_t_ versus t^0.5^. The multi-linearity in Fig. 9c indicated that the adsorption process took place in three stages as well as the multistage sorption of Cr(VI) on the NZVI-Si surface. The first stage describes the transfer of Cr(VI) from the solution to NZVI-Si outer surface or boundary layer diffusion. The second stage represents the entrance of Cr(VI) ions into the pores by intraparticle diffusion. The third stage refers to the diffusion of Cr(VI) into the small pores till reaching the equilibrium. The lines in Fig. 9c did not pass through the origin affirming that the adsorption of Cr(VI) could be attained via intraparticle diffusion but it was not the only rate-governing step^56^. Moreover, the non-zero intercept in the case of the Boyd model in Fig. 9d reaffirmed that the rate of adsorption was controlled by intraparticle diffusion. The high R^2^ of the parabolic diffusion model affirmed that the adsorption of Cr(VI) could occur by intraparticle diffusion as shown in Fig. 9e. Linear plot of q_t_ versus ln(t) as depicted in Fig. 9f was used to estimate the Elovich model constants and R^2^. The high R^2^ indicated that Elovich model was satisfactory to describe the adsorption process. Power kinetic model constants were determined via the linear plot of ln(q_t_) versus ln(t) and the modest R^2^ value expressed that this model could not effectively describe the adsorption process (Fig. 9g). Table 1 shows the kinetic models’ constants and R^2^.

**Table 1:** Constants and coefficients of determination of pseudo-first-order, pseudo-second-order, Elovich, Boyd, parabolic diffusion and power function kinetic models.

Kinetic models	Parameters
First-order model	K_1_ = 0.1222 min^-1^ q_e_ = 18.04 mg g^-1^ R^2^ = 0.9714
Second-order model	K_2_ = 0.0088 g mg^-1^ min^-1^ q_e_ = 24.8 mg g^-1^ R^2^ = .9972
Elovich model	α = 17.23 mg g^-1^ min^-1^ β = 0.195 g mg^-1^ R^2^ = 0.9698
Intraparticle diffusion model	K_d_ = 0.5454 mg g^-1^ min^-0.5^ C = 19.358 mg g^-1^ R^2^ = 0.9381
Boyd model	R^2^ = 0.9832
Parabolic diffusion model	K_p_ = 0.5934 (mg kg^-1^)^-0.5^ a = 18.247 mg g^-1^ R^2^ = 0.9516
Power function model	a = 205 b = 0.222 R^2^ = 0.8359

### Adsorption isotherms

**Figure 10:**
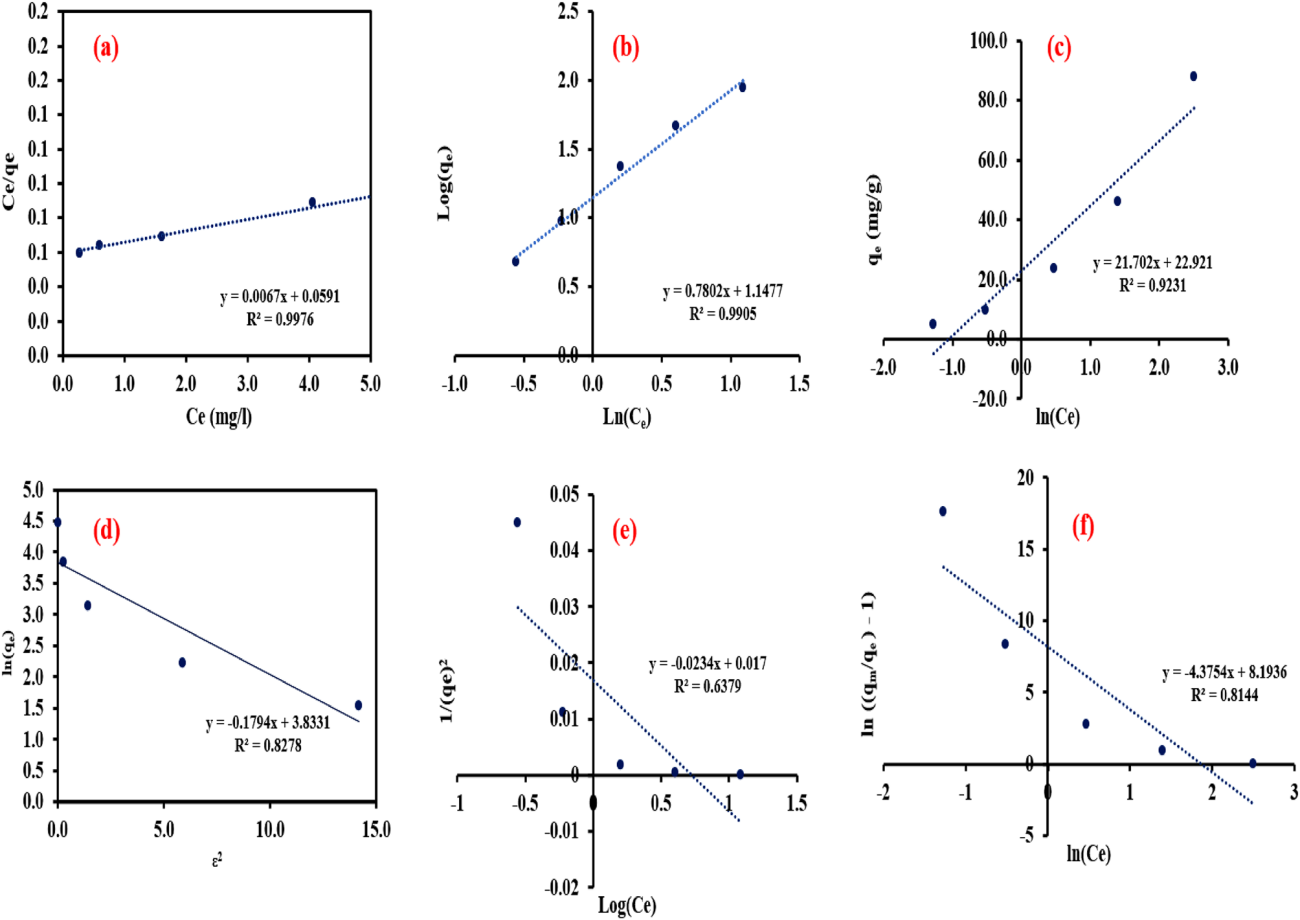
Adsorption isotherms (**a**) Langmuir model, (**b**) Freundlich model, (**c**) Temkin model, (**d**) Dubinin-Radushkevish model, (**e**) Harkins–Jura model and (**f**) Generalized isotherm model.

The linear relation between C_e_ and C_e_/q_e_ was plotted to estimate the Langmuir isotherm constants and R^2^ as portrayed in Fig. 10a. The high R^2^ (0.9976) indicated that the adsorption isotherm of Cr(VI) on the adsorbent’s surface could be expressed by the Langmuir model. The maximum monolayer adsorption capacity was 149.25 mg g^−1^. R_L_ value was lower than 1 confirming the favorable adsorption of Cr(VI) on NZVI-Si surface confirming the suggested mechanism for Cr(VI) removal. Moreover, Freundlich and Temkin isotherm models’ constants were estimated from the slope and intercept of the linear plots shown in Fig. 10b,c. The parameters of the different studied equilibrium isotherm models are listed in Table 2. The R^2^ was lower in the case of Freundlich and Temkin indicating that Langmuir was more suitable to fit the experimental data. The value of 1/n in Freundlich equation was lower than 1 suggesting the favorability of the adsorption of Cr(VI) on the NZVI-Si surface. The linear plot of ε^2^ versus ln(q_e_) could be employed to estimate Dubinin-Radushkevish constants (Fig. 10d). The mean adsorption energy calculated as 17.5 kJ mol^-1^ indicating that the adsorption of Cr(VI) on NZVI-Si surface is physico-chemical adsorption. The low R^2^ in the Hankins-Jura model is as shown in Fig. 10e depicted that the adsorption process was monolayer which was in agreement with the results obtained from Langmuir model. The Generalized isotherm model constants were estimated via the linear plot of ln($\frac{{q}_{m}}{{q}_{e}}-1)$ versus ln(C_e_). The generalized isotherm model could not describe the adsorption process of Cr(VI) on NZVI-Si because of its low R^2^ compared to other isotherm models (Fig. 10f).

**Table 2:** Constants and coefficients of determination of isotherm models.

Isotherm models	Parameters
Langmuir model	q_m_ = 149.25 mg g^-1^ K_L_ = 0.113 L mg^-1^ R^2^ = 0.9976 R_L_ = 0.0815
Freundlich model	K_f_ = 3.151 L mg^-1^ 1/n = 0.78 R^2^ = 0.99
Temkin model	b_t_ = 112.875 J mol^-1^ k_t_ = 2.875 L mg^-1^ B = 112.3 R^2^ = 0.9231
Dubinin-Radushevish model	Qs = 44.4 mg g^-1^ B = 0.1635 mol^2^ J^-2^ R^2^ = 0.9667 E = 17.5 kJ mol^-1^
Harkins–Jura model	A = 42.7 B = 0.73 R^2^ = 0.6379
Generalized isotherm model	K = 3617.7 N_b_ = 4.3754 R^2^ = 0.8144

### Adsorption thermodynamics

**Figure 11:**
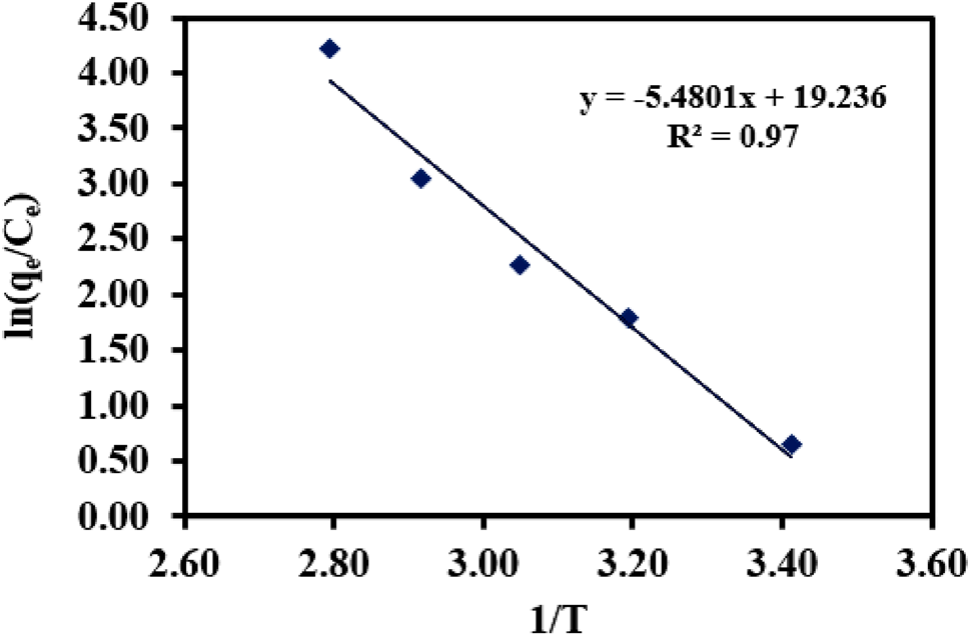
Linear plot of ln(q_e_/C_e_) versus 1/T.

The linear plot of ln(q_e_/q_c_) versus 1/T is shown in Fig. 11. ΔH^o^, ΔS^o^ and ΔG^o^ values were estimated and listed in Table 3. The negative value of ΔG^o^ and negative value of ΔH^o^ suggested the spontaneous and exothermic nature of Cr(VI) adsorption on the NZVI-Si surface. Moreover, the positive value of ΔS^o^ indicated that the randomness at the sorbent/solution interface was high. The increase of ΔG^o^ with raising the temperature reflected the increase of spontaneity of the adsorption process with temperature.

**Table 3:** Parameters of thermodynamic study.

Temperature (K)	ΔG^o^ (KJ mol^-1^)	ΔH^o^ (KJ mol^-1^)	ΔS^o^ (J mol^-1^ K^-1^)
293	-1.3	-45.56	159.93
313	-4.5		
328	-6.9		
343	-9.3		
358	-11.69		

### Reusability of NZVI/Si

**Figure 12:**
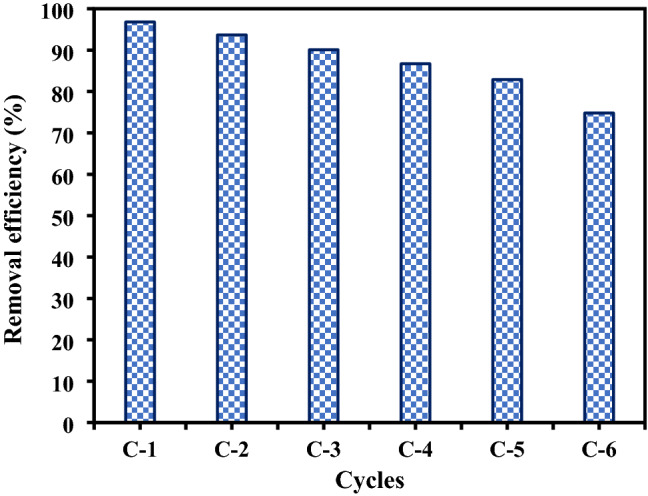
Reusability performance of NZVI-Si at NZVI-Si dose of 0.1 g/100 mL, initial Cr(VI) concentration of 25 mg/L, contact time of 120 min, and temperature of 20 °C.

NZVI-Si was used in repetitive cycles to investigate its reusability performance as shown in Fig. 12. The NZVI-Si particles were collected after each cycle by a magnet, washed with water and dried before the subsequent use. The removal efficiencies were 96.8%, 93.67%, 90.1%, 86.7, 82.9% and 74.8% in the six consecutive runs, respectively. The results indicated the efficient reusability of NZVI-Si. The removal efficiency was higher in the first cycle owing to the availability of active sites. However, the number of binding sites decreased in the following runs. Moreover, the removal efficiency decreased in successive cycles due to the oxidation of NZVI-Si and the formation of a passivation layer in successive cycles^8^.

### Removal mechanism of Cr(VI) by NZVI-Si

**Figure 13:**
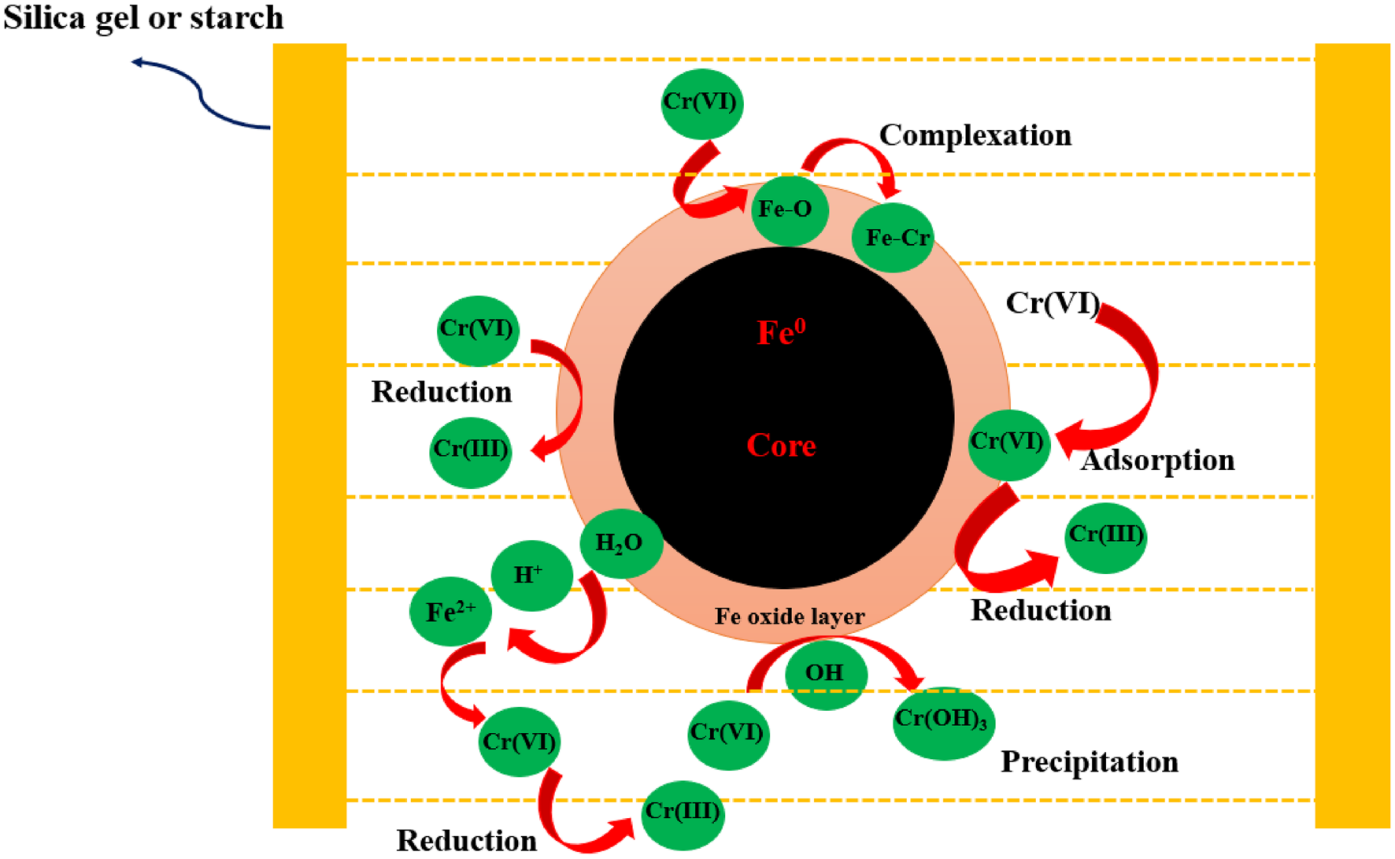
Removal mechanism of Cr(VI) by supported-NZVI.

Figure 13 shows the three-step removal mechanism of Cr(VI) (adsorption, reduction and precipitation). NZVI nanoparticles consist of a core and shell. The shell is composed of iron oxides that can be formed via the environmental oxidation of NZVI^51^. Moreover, NZVI can react with water and oxygen in aqueous solutions forming iron hydroxide layer on the NZVI surface^57^. Cr(VI) can be adsorbed on the NZVI surface, as Cr(VI) can form chemical complexes with iron oxide/hydroxide in the outer layer^58^. The NZVI can react with H_2_O generating Fe^2+^ and H^+^ (secondary reductants) as given in Eq. (3). Moreover, the iron oxide/hydroxide layer has a high reducing ability that can reduce the adsorbed Cr(VI) to Cr(III)^59^. Then, the Cr(OH)_3_ can be formed on the NZVI surface and then precipitated as shown in Eq. (4). Moreover, Cr^3+^ can be incorporated into the iron oxide/hydroxide layer forming Fe^3+^-Cr^3+^ complexes. The formed Fe^3+^-Cr^3+^ hydroxides can de-accelerate the electron transfer from the core to the surface which inhibits the reduction of Cr(VI) and reduces the removal performance especially at high initial Cr(VI) concentrations^59^. The adsorption of Cr(VI) on NZVI surface was confirmed by EDS, EDS elemental mapping and XPS. Moreover, XPS analysis affirmed the reduction of Cr(VI) to Cr(III).

The support on silica or starch can facilitate the electron transfer and inhibit the rapid oxidation of NZVI surface which improves the removal of Cr(VI)^50^. Moreover, supporting on silica or starch can decrease the agglomeration of NZVI which improves the reactivity, reducibility and dispersibility of NZVI as shown in TEM images. Further, the Cr(VI) can diffuse into the pores of the supporting materials as well as supporting materials can provide NZVI with higher surface area and active sites which increased the adsorption and reduction performance of bare NZVI^56^. Additionally, the starch or silica can employ as nanoreactors for accelerating the reaction between supported-NZVI and Cr(VI) and the NZVI in the pores can effectively reduce Cr(VI)^60^. The difference between Cr(VI) concentration in the outer and inner pores creates a driving force that participates in attaining frequent adsorption and diffusion of Cr(VI) into the pores till reaching the equilibrium^61^. Thus, the supported-NZVI can effectively remove Cr(VI).

3 $$ {\text{Fe}}^{0} + {\text{ 2 H}}_{{2}} {\text{O}} \to {\text{Fe}}^{{{2} + }} + {\text{ H}}_{{2}} + {\text{ 2 OH}}^{ - } $$

4 $$ {\text{2 Fe}}^{0} + {\text{ Cr}}_{{2}} {\text{O}}_{{7}}^{{{2} - }} + {\text{ 8 H}}^{ + } \to {\text{3 Fe}}^{{{2} + }} + {\text{ 2 Cr}}\left( {{\text{OH}}} \right)_{{3}} + {\text{ H}}_{{2}} {\text{O}} $$

### Role of silica in the removal of Cr (VI)

From the abovementioned data, the silica supported Fe^0^ is faster than that of pure Fe^0^. This may be one of three mechanisms which are (i) adsorptive behavior of silica on the surface of Fe^0^; (ii) silica as a governer of formation of Fe^0^ and possessed a tail-loop surface. Cr(VI) is diffused into the position of tail-loop layers and the availability of Cr(VI) on the surface of Fe^0^ would be increased; and (iii) Protection of Fe^0^ by silica to provide more contact area of Cr(VI) as a target pollutants.

## Materials and methods

### Materials

Ferrous chloride (FeCl_2_, 99%), ferrous chloride tetrahydrate (FeCl_2_.4H_2_O, 99%), ferrous sulfate heptahydrate (FeSO_4_.7H_2_O, 99%), ferric chloride hexahydrate (FeCl_3_.6H_2_O, 99%), sodium borohydride (NaBH_4_, 99%) and hydrochloric acid (HCl, 99%) were purchased from Sigma-Aldrich. Ethanol (C_2_H_5_OH, 99%), sodium hydroxide (NaOH, 33%), hydrazine (N_2_H_4_, 99%) and ammonium hydroxide (NH_4_OH, 99%) were procured from Pharos company (Egypt). Silica gel (SiO_2_, 99%) was purchased from Fluka in Switzerland and starch (99%) was procured from Nice Chemicals in India. Potassium dichromate (K_2_Cr_2_O_7_, 97%) and humic acid (C_9_H_9_NO_6_, 99%) were obtained from Sigma-Aldrich. Zinc chloride (ZnCl_2_, 97%), magnesium chloride hexahydrate (MgCl_2_.6H_2_O, 99%), sodium nitrate (NaNO_3_, 98%), copper chloride dihydrate (CuCl_2_.2H_2_O, 99%), sodium sulfate (Na_2_SO_4_, 99%), sodium phosphate dodecahydrate (Na_3_PO_4_.12H_2_O, 99%) and sodium carbonate dehydrate (Na_2_CO_3_.10H_2_O, 98%) were purchased from Loba Chemie (India). All chemicals were of high purity and used directly without any modifications.

### Preparation of pure nano zero-valent iron (NZVI) and modified NZVI composite

NZVI was synthesized via the liquid-phase reduction method in which ferrous ions (Fe^2+^) in an aqueous solution can be rapidly reduced to zero-valent ion (Fe^0^) using sodium borohydride (NaBH_4_) as a reducing agent as shown in Eq. (5). The optimum reaction time, reducing agent and iron salt were specified based on the results of the optimization of NZVI preparation process that were discussed in the results and discussion section.

5 $$  {\text{Fe}}^{{{\text{2}} + }} _{{({\text{aq}})}}  + {\text{ 2BH}}_{{4({\text{aq}})}}^{ - }  + {\text{ 6H}}_{{\text{2}}} {\text{O }} \to {\text{ Fe}}^{0} _{{({\text{s}})}}  + {\text{ 2B}}\left( {{\text{OH}}} \right)_{{{\text{3}}({\text{aq}})}}  + {\text{ 7H}}_{{\text{2}}}  \uparrow  $$

In detail, 1 M of FeCl_2_.4H_2_O was added to 50 mL of ethanol. Subsequently, 4 M of NaBH_4_ was mixed with 20 mL of distilled water and the formed solution was added dropwise (50–60 drops/min) to the ferrous solution during mixing in an aerobic condition (Without purging N_2_ or Ar). The mixture color turned to black after adding the NaBH_4_ solution affirming the reduction of ferrous ions to zero-valent iron and the mixture was further stirred for 10 min to secure the time required for the complete reduction of ferrous ions to Fe^0^. Then, the particles were collected using an external magnet and washed five times with distilled water and absolute ethanol to ensure the removal of reducing agent residuals. After washing, the nanoparticles were collected by centrifugation at 6000 rpm followed by drying at 50 °C overnight in a vacuum condition.

To prepare the NZVI based composite nanomaterials through supporting on either organic starch (NZVI-St) or inorganic silica gel (NZVI-Si), the same procedures for the preparation of the bare NZVI were followed beside the addition of ferrous solution and ethanol to 0.3 g of silica gel or starch and sonication for 30 min to affirm the dispersion of supporting materials before the addition of NaBH_4_ solution.

### Experimental procedures

The preparation of NZVI was conducted at reaction times of 10, 30, 60 and 120 min using different iron salts and reducing agents under vigorous stirring. The yield of the prepared NZVI samples was estimated and the performance of the prepared NZVI for the removal of acid blue-25 was evaluated at initial dye concentration of 50 mg L^-1^, NZVI dose of 0.1 g, solution volume of 100 mL, contact time of 60 min and agitation rate of 200 rpm. The synthesis of NZVI supported on the surface of silica or starch was performed by adding the iron solution (FeCl_2_.4H_2_O) and ethanol on the supporting material. Then, the reducing agent (sodium borohydride (NaBH_4_)) was added to reduce iron ions to zero-valent iron supported on silica or starch. The adsorption integrated with chemical reduction of chromium Cr(VI) or acid blue-25 by the synthesized nanoparticles was conducted in a screw cap glass bottle. The bottle was filled with 100 mL of Cr(VI) or acid blue-25 dye solution and 0.1 g of the adsorbent was added at 20 °C. Then, the contaminated solution with the added adsorbent was placed in an incubator shaker for 120 min to remove Cr(VI) and 60 min for the removal of acid blue-25 (200 rpm). A stock solution of Cr(VI)) with a concentration of 1000 mg L^-1^ was prepared by dissolving 0.2829 g of K_2_Cr_2_O_7_ in distilled water (1000 mL) and another stock solution of acid blue-25 dye was prepared. The pH values were adjusted using 0.1 M of NaOH or HCl and the effect of pH (1–11) was investigated as well as the effect of initial Cr(VI) concentration (5–100 mg L^−1^) on the removal efficiency was studied at an adsorbent dose of 0.1 g, solution volume of 100 mL, contact time of 120 min, temperature of 20 °C and agitation rate of 200 rpm. The effects of the existence of cations such as Mg^2+^, Zn^2+^ and Cu^2+^, anions viz., NO_3_^-^, CO_3_^2-^, SO_4_^2-^ and PO_4_^3-^ and humic acid on the removal efficacy were investigated. The concentration of anions and cations was 40 mg L^−1^ and the investigation of the effect of humic acid on the adsorption system was conducted using two concentrations of humic acid (5 mg L^−1^ and 20 mg L^−1^) at NZVI-Si dose of 0.1 g/100 mL, pH 1, initial Cr(VI) concentration of 25 mg L^−1^, contact time of 120 min and temperature of 20 °C. The reusability study was conducted for six cycles at NZVI-Si dose of 0.1 g/100 mL, initial Cr(VI) concentration of 25 mg/L, pH 1, contact time of 120 min, and temperature of 20 °C. The particles were collected after each cycle using a magnet, and then they were washed with water and dried for successive use. The effect of adding H_2_O_2_ (0.25–1 mM) on the removal performance was investigated at NZVI-Si dose of 0.1 g/100 mL, initial Cr(VI) concentration of 25 mg L^−1^, contact time of 120 min and temperature of 20 °C. The samples were withdrawn during the contact time and centrifuged to separate the nanoparticles. Thereafter, measurement of Cr(VI) and acid blue-25 concentrations was performed using a UV spectrophotometer instrument (JASCO V-630) at 540 and 602 nm, respectively. Adsorption kinetics were studied during the time interval (0–120 min) at an initial Cr(VI) concentration of 25 mg L^−1^ and pH 1 using first-order, second-order, intraparticle diffusion, Elovich, power function, parabolic diffusion and Boyd kinetic models and investigation of the adsorption equilibrium was conducted at initial Cr(VI) concentrations of 5, 10, 25, 50 and 100 mg L^−1^, pH 1 and time of 120 min using Langmuir, Freundlich, Dubinin-Radushkevish, Temkin, Harkins–Jura and Generalized isotherm models. Thermodynamic study was performed at different temperatures (293, 313, 328, 343 and 358 K). The equations and discussion of kinetic, thermodynamic and isotherm models were provided in the supplementary file (Text S1).

The removal percentage of Cr(VI) or acid blue-25 dye was calculated as shown in Eq. (6):

6 $$ {\text{Removal }}\% \, = \, \left( {\left( {C_{{\text{o}}} {-}C_{{\text{e}}} } \right)/C_{{\text{o}}} } \right) \, *{1}00 $$

where *C*_o_ is the initial Cr(VI) or dye concentration (mg L^−1^) and *C*_e_ is the Cr(VI) or dye concentration at equilibrium (mg L^−1^). The adsorption capacities of the synthesized materials were calculated using Eq. (7):

7 $$ {\text{q}}_{{\text{e}}} = {\text{ V }}\left( {{\text{C}}_{{\text{o}}} {-}{\text{ C}}_{{\text{e}}} } \right)/{\text{m}} $$

where q_e_ is the adsorption capacity of the synthesized materials at equilibrium (mg g^-1^); V is the solution volume (L); and m is the mass of synthesized nanomaterials (g).

### Analytical methods

The diffraction planes of the synthesized nanomaterials were specified using X-ray diffraction analysis (XRD, Siemens model D-5000 diffractometer). The morphology, crystallinity, and chemical composition of the synthesized materials were investigated by performing transmission electron microscopy (TEM) coupled with energy dispersive X-ray spectroscopy (EDS), elemental mapping and selected area electron diffraction (SAED) (JEOL JEM-2100). The chemical bonds existing in the synthesized nanomaterials were specified using Fourier transform infrared spectroscopy (Shimadzu, FTIR-8400S). The surface area and pore size distribution of the synthesized nanomaterials were estimated using Belsorp-max automated apparatus (BEL Japan). Moreover, the chemical composition and oxidation states of the prepared nanoparticles were studied by performing an X-ray photoelectron spectroscopy analysis (Thermo-Fisher, USA). The magnetic characteristics of the synthesized nanomaterials were evaluated using vibrating sample magnetometer (VSM, Lake Shore-7410, USA), magnetic field up to 20 kOe and the sensitivity up to 1 μ emu. The point of zero charge was estimated using solid addition method as reported in our previous work^56^.

## Conclusions

Sodium borohydride and ferrous chloride tetrahydrate were the optimum reducing agent and iron precursor, respectively for the preparation of pure NZVI after a reaction time of 10 min. The NZVI surface was modified it by supporting on starch or silica gel. The excellent support on modifiers was confirmed by various analyses such as TEM, EDS and XPS. The optimized NZVI can attain full removal of acid blue-25 dye after 60 min. NZVI-Si showed higher performance than pure NZVI and NZVI-St. The adsorption capacity was improved at elevated concentrations of Cr(VI) under acidic conditions. Adsorption kinetics, isotherms and thermodynamics studies indicated that the adsorption process was physical, favorable, spontaneous and endothermic. The existence of cations such as Mg^2+^, Zn^2+^ and Cu^2+^ and anions like NO_3_^-^, CO_3_^2-^, SO_4_^2-^, PO_4_^3-^ decreased the removal efficiency of Cr(VI). The addition of alow concentration of HA (5 mg L^−1^) can improve the removal efficiency compared to reduced removal performance at high HA concentration (10 mg L^−1^). The addition of H_2_O_2_ with a concentration over 0.75 mM reduced the removal efficiency. The removal efficiencies were 96.8%, 93.67%, 90.1%, 86.7, 82.9% and 74.8% after six repetitive cycles using NZVI-Si. Reduction, adsorption and precipitation were the major removal Cr(VI) mechanisms onto the prepared NZVI-Si.

## Supplementary Information

Supplementary Information.

## Data Availability

The data that support the findings of this study are available from the corresponding author upon reasonable request.
