# Supplementary material for: The superior performance of silica gel supported nano zero-valent iron for simultaneous removal of Cr (VI)
Source: Sci Rep. 2022 Dec 23;12:22443. doi: 10.1038/s41598-022-26612-1 (PMC9794730; doi:10.1038/s41598-022-26612-1)
Supplement: Supplementary file 1 — Supplementary Information. [file 41598_2022_26612_MOESM1_ESM.docx]

**The superior performance of silica gel supported nano zero-valent iron for simultaneous removal of Cr (VI)**

Eslam Salama^1^, Mahmoud Samy^2^, Hassan Shokry^3,4^, Gehan El-Subruiti^5^, Asmaa El-Sharkawy^5^, Hesham Hamad^6,*^, Marwa Elkady^6,7, *^

^1^ Environment and Natural Materials Research Institute (ENMRI), City of Scientific Research and Technological Applications (SRTA-City), New Borg El-Arab City, 21934, Alexandria, Egypt

^2^ Department of Public Works Engineering, Faculty of Engineering, Mansoura University, Mansoura, 35516, Egypt

^3^ Environmental Engineering Department, Egypt-Japan University of Science and Technology (E-JUST), New Borg El-Arab City, Alexandria, Egypt

^4^ Electronic Materials Research Department, Advanced Technology and New Materials Research Institute, City of Scientific Research and Technological Applications (SRTA-City), New Borg El-Arab City, 21934, Alexandria, Egypt

^5^ Chemistry Department, Faculty of Science, Alexandria University, Ibrahimia, Alexandria, Egypt

^6^ Fabrication Technology Research Department, Advanced Technology and New Materials Research Institute, City of Scientific Research and Technological Applications (SRTA-City), New Borg El-Arab City, 21934 Alexandria, Egypt.

^7^ Chemical and Petrochemical Engineering Department, Egypt-Japan University of Science and Technology (E-JUST), New Borg El-Arab City, Alexandria 21934, Egypt

*****Corresponding Author.

Email address: H. Hamad: [heshamaterials@hotmail.com](mailto:heshamaterials@hotmail.com), hhamad@srtacity.sci.eg

Email address: M.F. Elkady: [marwa.f.elkady@gmail.com](mailto:marwa.f.elkady@gmail.com)

**Text S1**

**a. Adsorption kinetics**

The adsorption mechanism of Cr(VI) on the adsorbent’s surface was investigated and the experimental data were fitted using various models such as pseudo-first-order, pseudo-second-order, intraparticle diffusion, Elovich and Boyd. Moreover, the adsorption rates were estimated using the aforementioned models.

In pseudo-first-order model, the adsorption rate is proportional to the vacant adsorption sites. Eq. (1) shows the pseudo-first-order model.

Ln(q_e_-q_t_) = ln(q_e_) - K_1_ t (1)

where q_e_ and q_t_ are the uptake of the adsorbents at equillibrium and time (t), respectively. K_1_ is the pseudo-first-order rate constant (min^-1^).

The adsorption rate is propotional to the square of the number of unfilled adsorption sites in pseudo-second-order model and pseudo-second-order model is shown in Eq. (2) .

$\frac{t}{q_{t}}= \frac{1}{K_{2}}\frac{1}{{q_{e}}^{2}}+ \frac{t}{q_{e}}$ (2)

where k_2_ is the pseudo-second-order rate constant (g mg^-1^ min^-1^)

The transfer of Cr(VI) from the bulk solution to the adsorption sites of the synthesized adsorbents is governed by external film diffusion or intraparticle diffusion. Therefore, the diffusion mechanism was investigated using intraparticle diffusion, parabolic diffusion and Boyd kinetic models in Eqs. (3, 4, 5), respectively.

q_t_ = K_d_ t^0.5^ + C (3)

q_t_ = a + k_p_ t^0.5^  (4)

B_t_ = -0.4977 – ln(1-F) (5)

where K_d_ is the intraparticle diffusion rate constant (mg g^-1^ min^-0.5^), C refers to the boundary layer thickness (mg g^-1^), kp is the diffusion rate constant (mg kg^-1^)^-0.5^ and F = $\frac{q_{t}}{q_{e}}$ where F is the fraction of solute adsorbed at any time.

Elovich kinetic model can be used to express the adsorption kinetics of Cr(VI) on the adsorbents’ surface as shown in Eq. (6).

q_t_ = $\frac{1}{\beta_{e}}$ ln(αβ_e_) + $\frac{1}{\beta_{e}}$ ln(t) (6)

where α is the initial adsorption rate (mg g^-1^ min^-1^) and β is a parameter related to the degree of surface coverage and activation energy (g mg^-1^).

Power function kinetic model can be used to describe the adsorption system of Cr(VI) on the adsorbents’ surface with time as shown in Eq. (7).

log($q_{t}$) = log(a) + b log(t) (7)

a and b are the power function model constants.

**b. Adsorption isotherms**

Adsorption isotherms were employed to measure the adsorption capacity of adsorbents and understand how the adsorption of Cr(VI) on the synthesized nanomaterials took place. Langmuir, Freundlich, Harkins-Jura, Temkin, Generalized and Dubinin-Radushkevish isotherm models were employed to fit the experimental data.

Langmuir isotherm model in Eq. (8) assumes that only one layer of the sorbate can be formed on the sorbent’s surface which contains unlimited number of similar sites with homogenous adsorption energy.

$\frac{C_{e}}{q_{e}}$ = $\frac{C_{e}}{q_{m}}+ \frac{1}{K_{L}q_{m}}$ (8)

where q_e_ is the amount of Cr(VI) adsorbed per 1.0 g of the adsorbent at equillibrium (mg g^-1^), q_m_ is the maximum amount of Cr(VI) adsorbed on unit mass of the adsorbent (mg g^-1^), K_L_ is the Langmuir constant (L mg^-1^) and C_e_ is the concentration of Cr(VI) at equillibrium (mg L^-1^).

Eq. (9) shows the equillibrium constant (R_L_) that can express the favorability of adsorption process.

R_L_ = $\frac{1}{1+ K_{L} C_{o}}$ (9)

where C_o_ is the initial concentration, R_L_ is the separation factor describing the isotherm type (linear isotherm at R_L_ = 1, irreversible at R_L_ = 0, favorable isotherm at R_L_ between 0 and 1, and unfavorable at R_L_ > 1).

Freundlich model describes the equilibrium between the adsorbent and adsorbate for heterogeneous sorbents in the case of multilayer sorption with non-uniform distribution of adsorption heat. Frenduilch isotherm model is shown in Eq. (10):

Ln(q_e_) = ln(K_f_) + $\frac{1}{n}$ln(C_e_) (10)

where K_f_ )L mg^-1^) and n are Frenduilch constants which are associated with adsorption capacity and intensity, respectively and 1/n expects the adsorption process strength (favorable isotherm at 0 < 1/n < 1, irreversible at 1/n = 0, and unfavorable at 1/n > 1).

Dubinin-Radushkevish (D-R) isotherm model (Eq. (11)) can be employed to investigate the adsorption mechanism and specify whether the adsorption occurs chemically or physically.

ln(q_e_ )= ln(Q_s_) – B ε^2^ (11)

where Q_s_ is the theoretical saturation capacity (mg g^-1^), B is the D–R model constant (mol^2^ J^-2^), ε is the Polanyi potential and can be estimated using Eq. (12).

ε = R T ln(1+$\frac{1}{C_{e}}$) (12)

The mean energy of adsorption, E (kJ mol^-1^), can be estimated using Eq. (13).

E = $\frac{1}{\sqrt{2B}}$ (13)

where R is the universal gas constant (8.314 J mol^-1^ K^-1^), T is the solution temperature (K).

Temkin isotherm model takes in consideration the interactions between Cr(VI) and the synthesized adsorbents. Also, it assumes that the raising of adsorbent coverage linearly decreases the adsorption heat. Eq. (14) describes the Temkin isotherm model.

q_e_ = $\frac{\mathrm{RT}}{b_{t}}$ ln(k_t_) + $\frac{\mathrm{RT}}{b_{t}}$ ln(_Ce_) (14)

B = $\frac{\mathrm{RT}}{b_{t}}$ (15)

where b_t_ is Temkin constant related to adsorption heat (J mol^-1^), K_t_ is the Temkin isotherm equilibrium binding constant (L mg^-1^).

Eqs. (16, 17) describe Harkins-Jura isotherm model and generalized isotherm model, respectively. Harkins-Jura model was employed to specify whether the adsorption is monolayer or multilayer.

$\frac{1}{q_{e}^{2}}$ = ($\frac{B}{A})-\left( \frac{1}{A} \right)\log(C_{e}$) (16)

Ln($\frac{q_{m}}{q_{e}}-1)$ = ln(K) – N_b_ ln($C_{e})$ (17)

where q_e_ is the uptake at equillibrium, C_e_ is the equillibrium concentration, A and B are Harkins-Jura constants, q_m_ is the maximum adsorption capacity, K and N_b_ are the constants of saturation and coordination bond, respectively.

**c. Adsorption thermodynamics**

Thermodynamic study was conducted at different temperatures (293, 313, 328, 343 and 358 K). The change in enthalpy (ΔH^o^), entropy (ΔS^o^) and energy (ΔG^o^) can be measured using Eqs. (18, 19).

Ln($\frac{q_{e}}{C_{e}}$) =$\frac{{\Delta S}^{o}}{R}$ -$\frac{{\Delta H}^{o}}{R T}$ (18)

ΔG^o^ = ΔH^o^ – T ΔS^o^  (19)


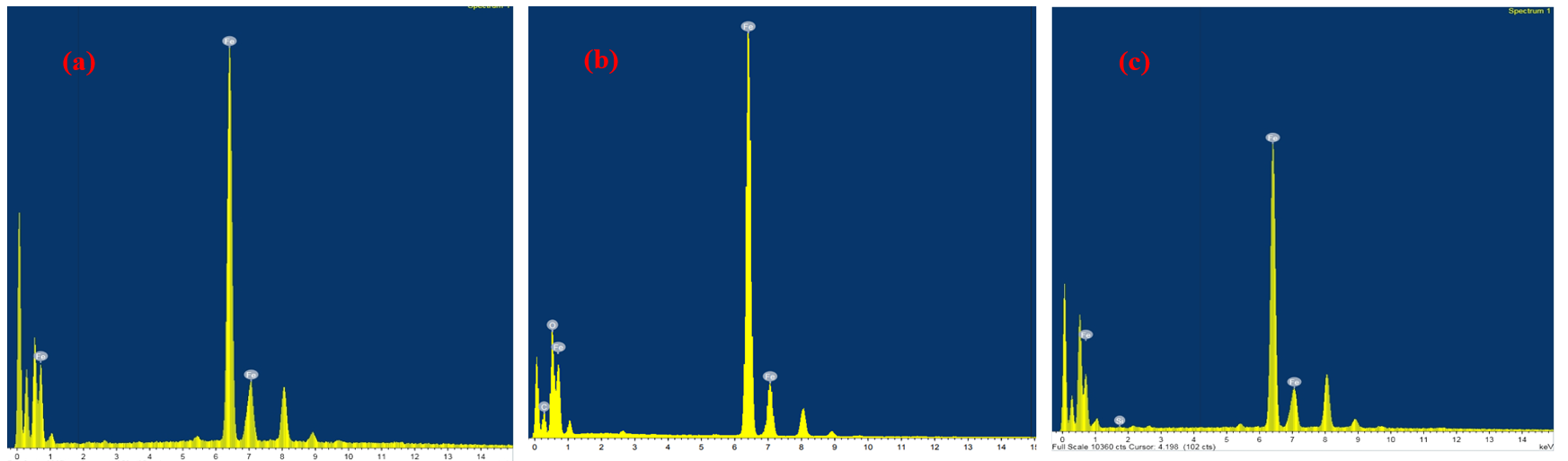


**Figure S1:** EDS patterns of (a) NZVI, (b) NZVI-St and (c) NZVI-Si.


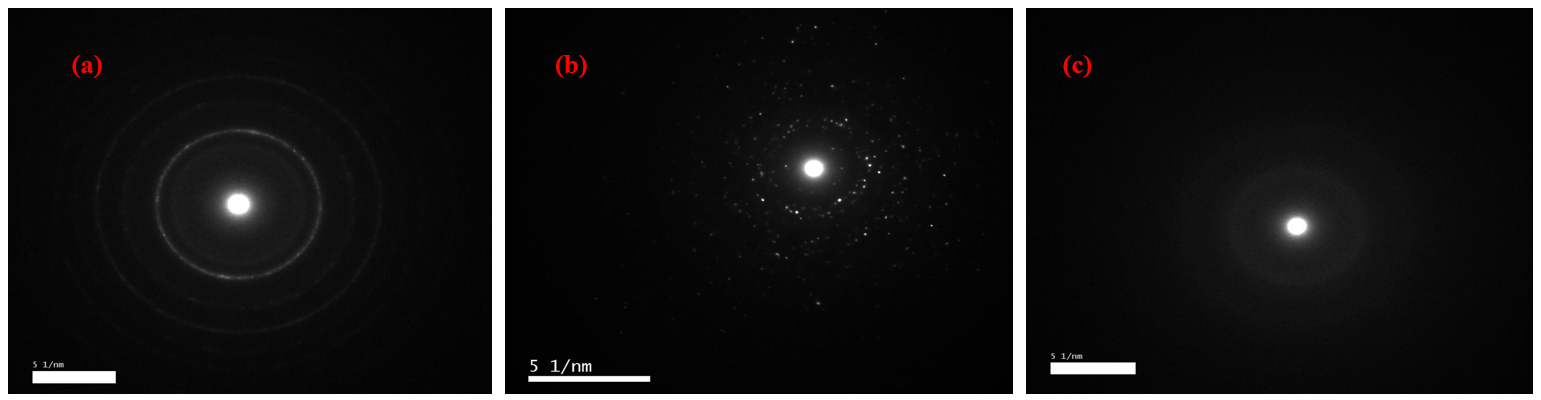


**Figure S2:** SAED patterns of (a) NZVI, (b) NZVI-St and (c) NZVI-Si.
